# Supplementary material for: In vivo CRISPR/Cas9 targeting of fusion oncogenes for selective elimination of cancer cells
Source: Nat Commun. 2020 Oct 8;11:5060. doi: 10.1038/s41467-020-18875-x (PMC7544871; doi:10.1038/s41467-020-18875-x)
Supplement: Supplementary file 1 — Supplementary Information [file 41467_2020_18875_MOESM1_ESM.pdf]

In vivo CRISPR/Cas9 targeting of fusion oncogenes for selective elimination of cancer cells

Martinez-Lage M#, Torres-Ruiz R#, et al.

Supplementary Information:

Content:

- Supplementary Figure 1
- Supplementary Figure 2
- Supplementary Figure 3
- Supplementary Figure 4
- Supplementary Figure 5
- Supplementary Figure 6
- Supplementary Figure 7
- Supplementary Figure 8

Corresponding authors:
Sandra Rodriguez-Perales
e-mail: srodriguezp@cnio.es
Raul Torres-Ruiz
e-mail: rtorres@carrerasresearch.org / rtorresr@cnio.es

Supplementary Figure 1

a

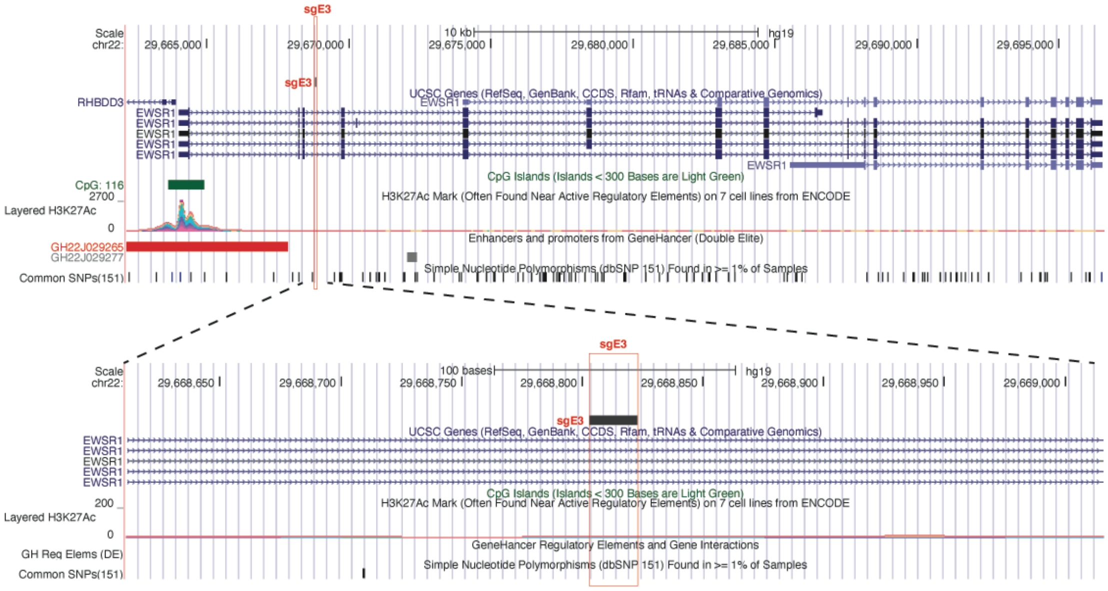

b

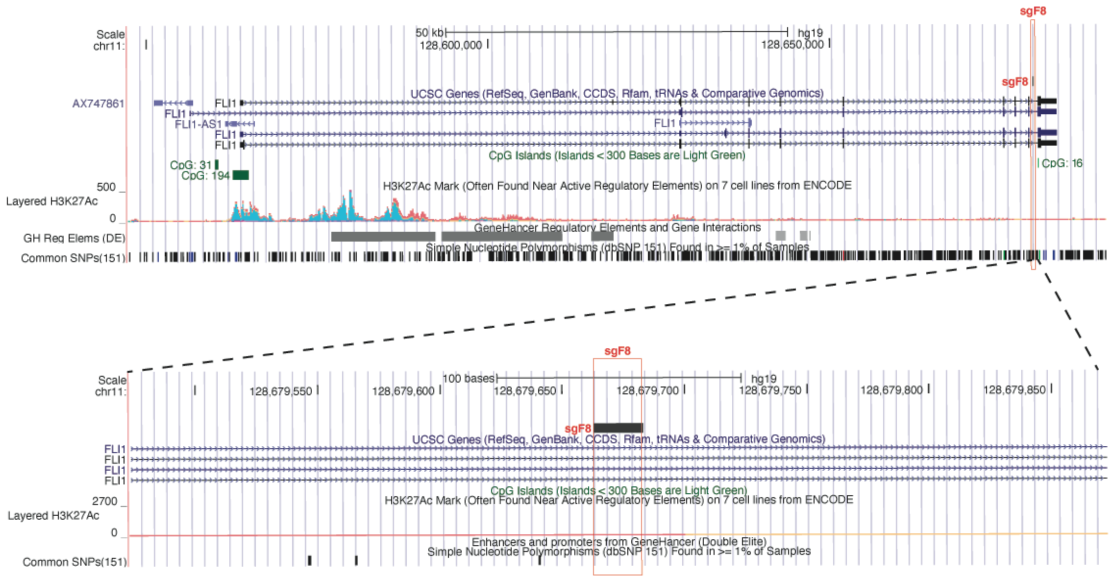

Supplementary Figure 1. USCC genome browser view of *EWSR1* and *FLI1* loci. a, Browser view of indicated regulation elements and common SNPs in the *EWSR1* gene (upper panel) and sgE3 (lower panel) targeted regions (genome.ucsc.edu). **b,** Browser view of indicated regulation elements and common SNPs in the *FLI1* gene (upper panel) and sgF8 (lower panel) targeted regions (genome.ucsc.edu).

Supplementary Figure 2

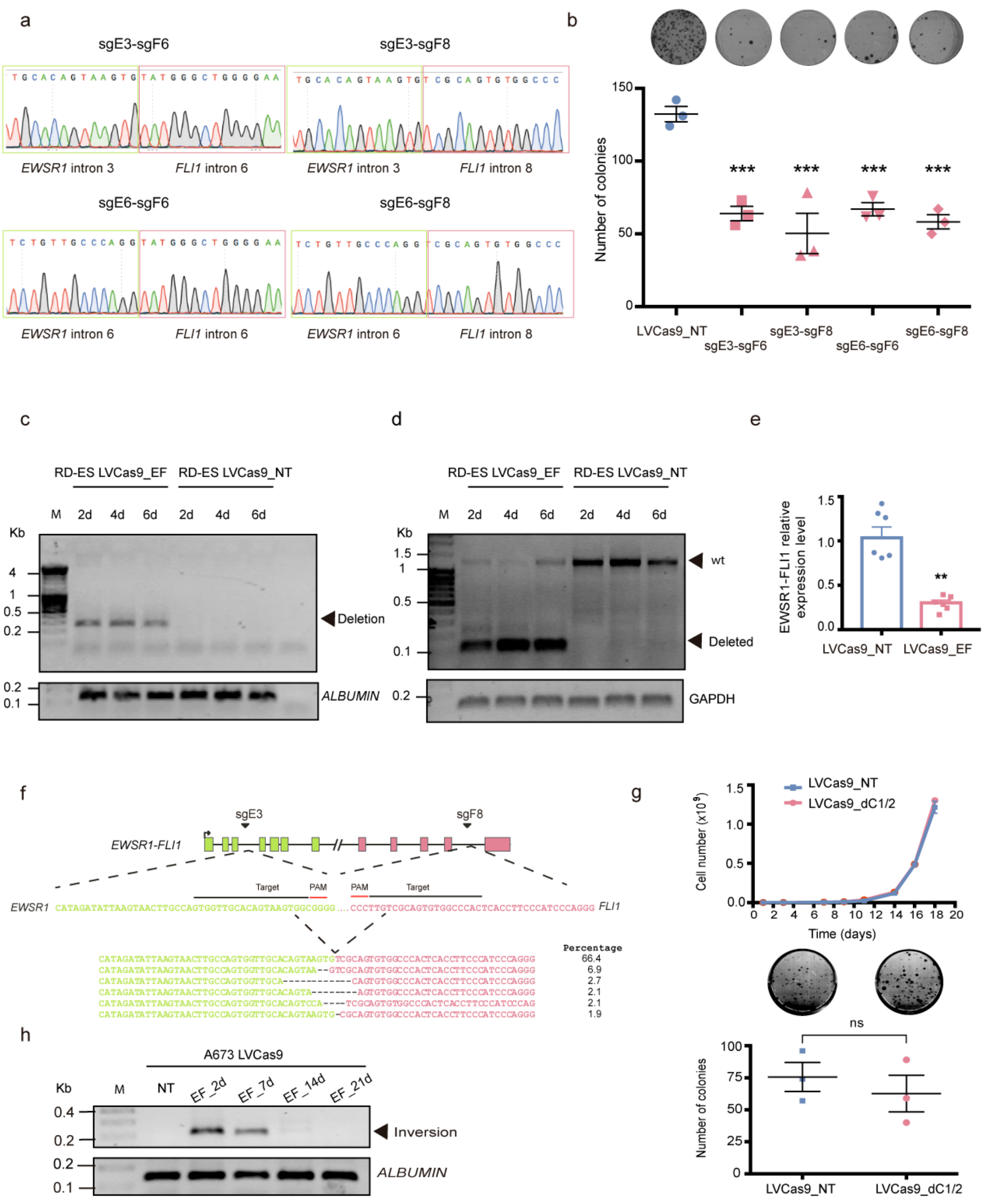

Supplementary Figure 2. Analysis of *EWSR1-FLI1* deletion. a, Sanger sequencing chromatograms showing the genomic PCR products analysis of edited A673 cells using oligos flanking the DNA loci targeted by the four sgRNA pair combinations. **b**, Colony formation assay representative images are shown for the A673 experimental and control cells. Graphical representation of the number of colonies formed in A673 control and edited cells, n=3 independent experiments (LVCas9_NT vs sgE3-sgF6 ***p=7e-4, LVCas9_NT vs sgE3-sgF8 ***p=0.001, LVCas9_NT vs sgE6-sgF6 ***p=7e-4, LVCas9_NT vs sgE6-sgF8 ***p=5e-4. **c**, Agarose gel electrophoresis showing the

DNA PCR analysis of edited and control RD-ES cell line using oligos flanking the DNA loci targeted by sgE3 and sgF8 (n=3, independent studies). The 300 bp PCR fragment denotes deletion of the DNA fragment. PCR analysis was done using DNA extracted from cells at day 2, 4 and 6 post-transduction (pt). *ALBUMIN* was used as an internal control of the PCR reaction. Bottom panel shows a representative chromatogram of Sanger sequencing analysis of the PCR products. **d**, Agarose gel electrophoresis of the *EWSR1-FLI1* RT-PCR products obtained from edited and control RD-ES cells (n=3, independent studies). RT-PCR analysis was done using RNA extracted from cells at day 2, 4 and 6 pt. Arrows depict the sizes of wild-type (1027 bp) and deleted (150 bp) RT-PCR products. *GAPDH* was used as an internal control of the RT-PCR reaction. Bottom panel shows a representative chromatogram of Sanger sequencing analysis of the RT-PCR products. **e,** *EWSR1-FLI1* expression level analysis. Relative expression level of *EWSR1-FLI1* in control (LVCas9_NT) and treated (LVCas9_EF) A673 cells measured by qRT-PCR and normalized to *GUSB*, n=6 independent experiments, **p=0.0013. **f**, Diagram and results of deep sequencing of the deletion PCR amplicons. **g**, Growth rate assay curve of A673 cells transduced with a non-targeting sgRNA control (LVCas9_NT) and dual control plasmid expressing two sgRNAs targeting two unrelated loci in two different chromosomes (LVCas9_dC1/2) cells (n=3), and representative crystal violet staining and statistical analysis of number of colonies (n=3). **h.** Agarose gel electrophoresis of the EWSR1-FLI1 inverted products of edited and control A673 cells (n=3, independent studies). Plot shows medians and ranges; error bars indicate the s.e.m. for the averages across the multiple experiments; p-values are represented (ns: non-significant, *p≤0.05, **p≤0.01, ***p≤0.001). PAM: protospacer adjacent motif. Two-tailed unpaired *t*-test was used for statistical analysis.

Supplementary Figure 3

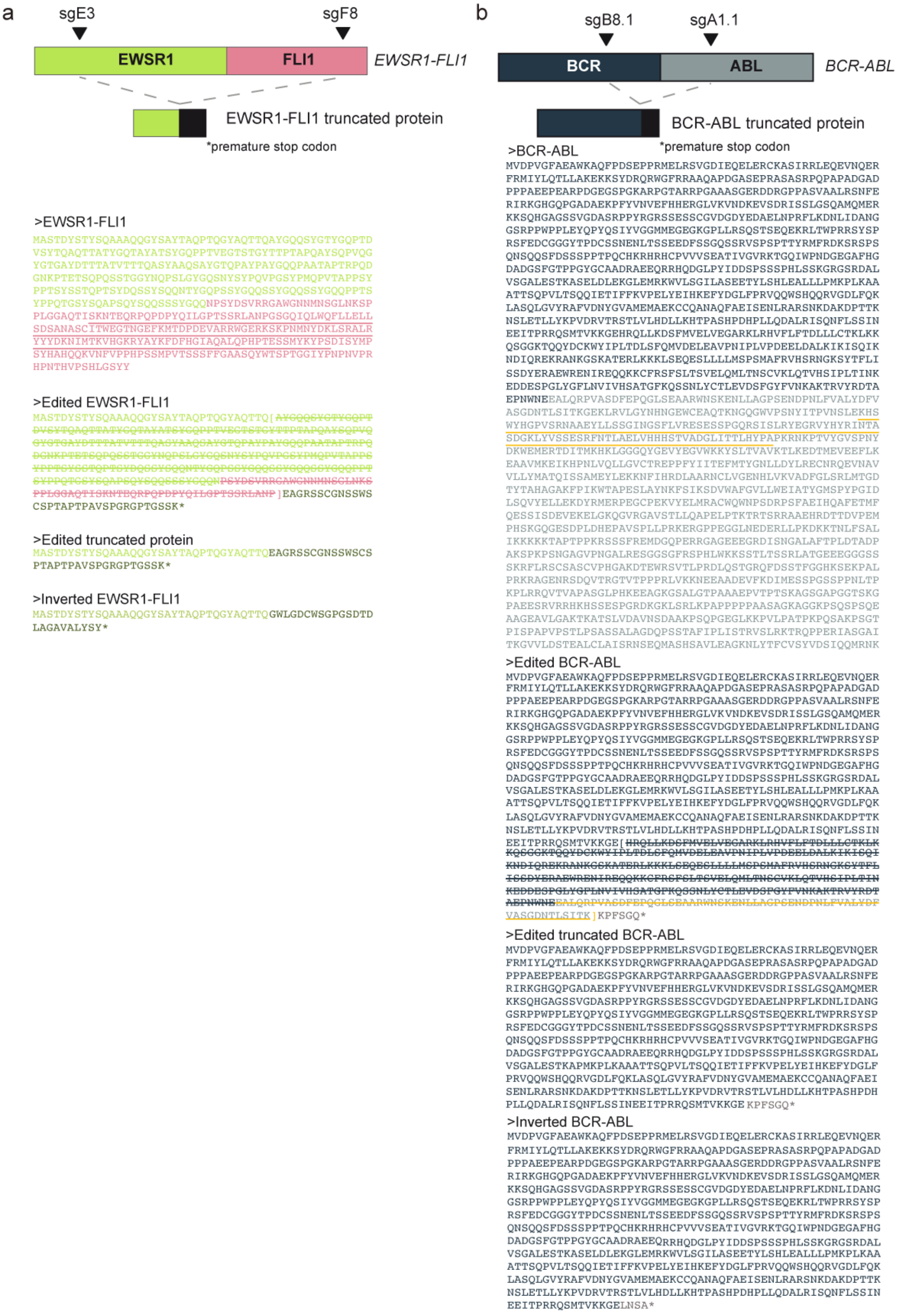

Supplementary Figure 3. EWSR1-FLI1 and BCR-ABL wild-type and edited predicted protein structures. a, Representative illustration of the EWSR1-FLI1

chimeric protein and truncated protein generated by genome editing deletion and inversion. Bottom panel shows amino acid sequence of the EWSR1-FLI1 protein (Type 1). Residues corresponding to EWSR1 or to FLI1 are shown in green or purple, respectively. EWSR1 transactivation and FLI1 DNA binding domains are underlined. Amino acid sequence of the edited EWSR1-FLI1 deleted and inverted proteins. Deleted residues are shown crossed out; the new residues generated by the change of reading frame after the mutation are shown in black. The premature STOP codon is shown with an asterisk. **b**, Amino acid sequence of the BCR-ABL1 protein (p210). Residues corresponding to BCR or to ABL1 are shown in black or grey, respectively. ABL1 DNA binding domain is underlined. Amino acid sequence of the edited BCR-ABL1 deleted and inverted proteins. Deleted residues are shown crossed out; the new residues generated by the change of reading frame after the mutation are shown in black. The premature STOP codon is shown with an asterisk.

Supplementary Figure 4

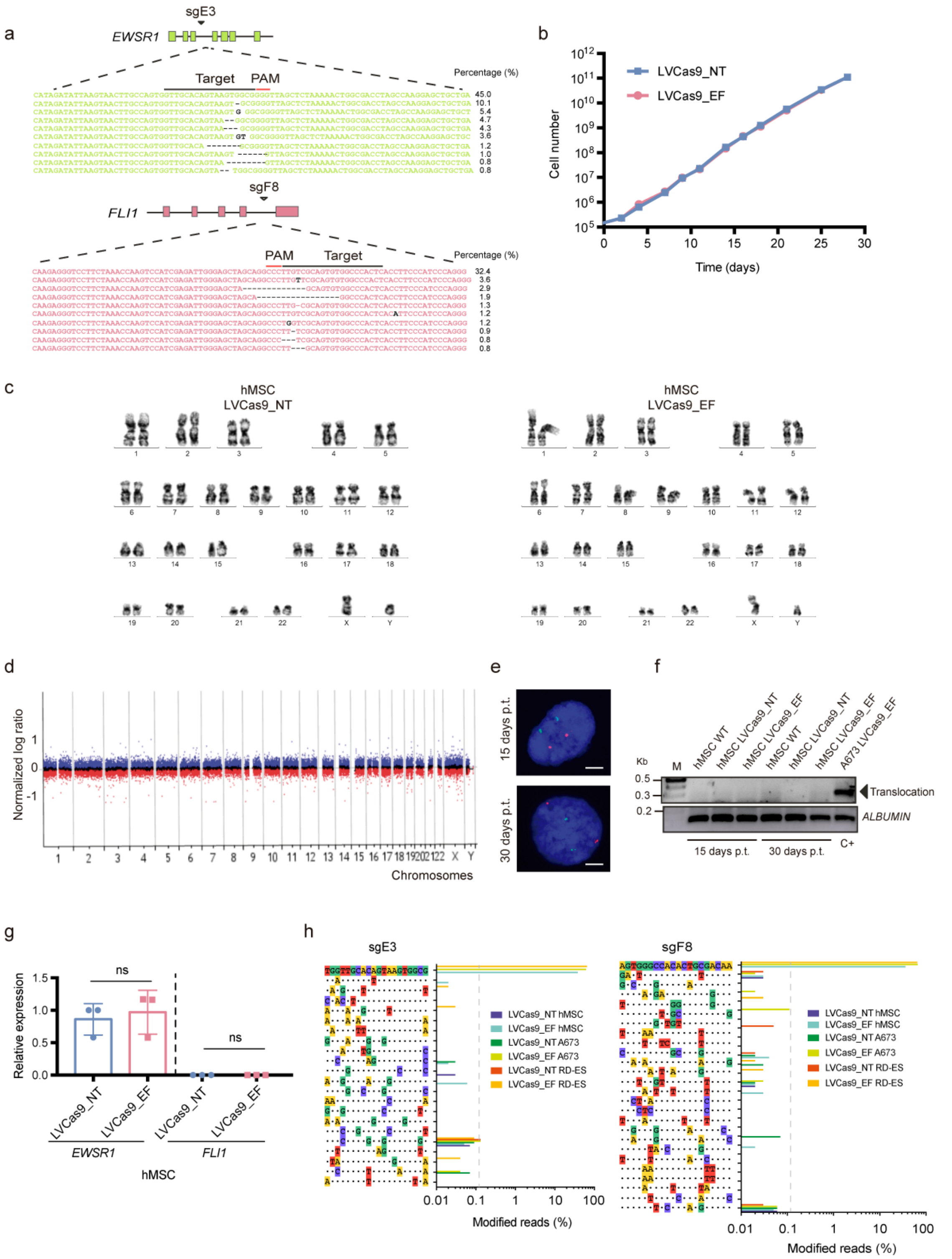

Supplementary Figure 4. Genome editing specificity analysis. a, Diagram and results of deep sequencing of the CRISPR-targeted *EWSR1* and *FLI1* PCR amplicons in hMSCs. **b,** Growth rate assay curve of hMSCs edited (LVCas9_EF) and control

(LVCas9_NT) cells (n=2). **c**, Representative G-banded metaphases with normal karyotypes of control and experimental hMSCs transduced with LVCas9_sgEF. **d**, Profile plot result of a high-density array comparative genomic hybridization (aCGH) analysis covering the whole genome, showing no copy number variations in hMSCs transduced with LVCas9_sgEF. Each dot represents a CGH-probe and zero value indicates equal fluorescence intensity ratio between the edited and control hMSCs. **e,** FISH (dual color dual fusion probe) analysis performed on LVCas9_EF treated hMSC showing representative interphase nuclei at days 15 and 30 p.t. (n=3, independent studies). The green signals represent the two normal chromosomes 22 (EWSR1 gene locus) and red signal the two normal chromosomes 11 (FLI1 gene locus). Scale bars, 5 um. **f,** PCR analysis to detect undesired translocations in wild type cells using oligos flanking the DNA targeted by sgE3 and sgF8 on LVCas9_NT and LVCas9_EF treated hMSC cells (n=3, independent studies). LVCas9_EF treated A673 cells are used as positive control. **g,** Transcriptional level of *EWSR1* and *FLI1* genes in edited hMSC (non-EWSR1-FLI1) control cells, n=3 independent experiments. The plots show medians and ranges; error bars indicate the s.e.m. for the averages across the multiple experiments; p-values are represented (ns: non-significant). **h**, Assessment of off-target indels determined by targeted amplicon sequencing. Colored boxes represent mismatches relative to the on-target sites. Bar graph shows a comparison of on- and off-target mutation rates. Two-tailed unpaired *t*-test was used for statistical analysis.

Supplementary Figure 5

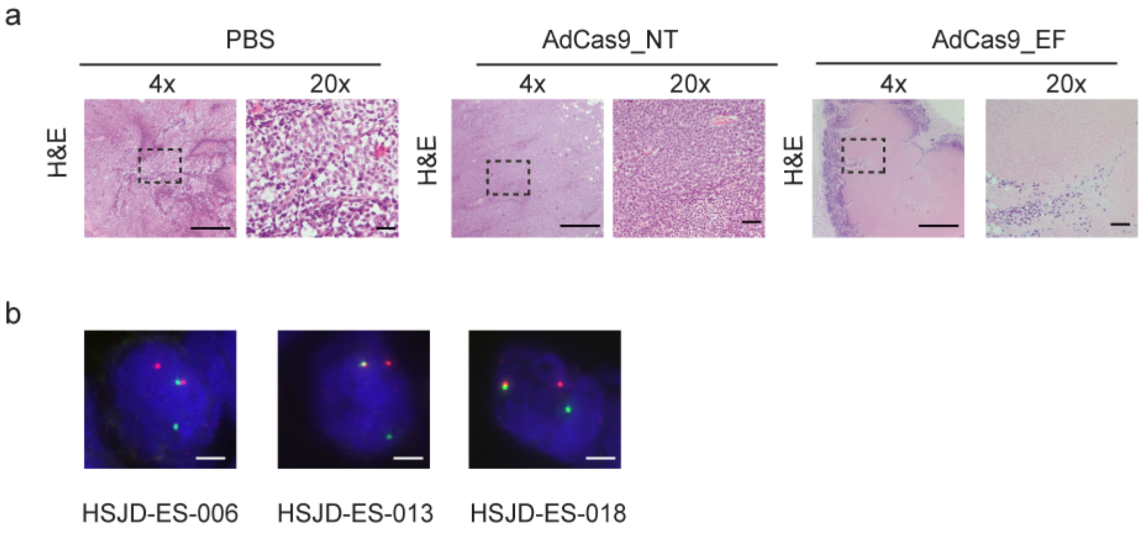

Supplementary Figure 5. Immunohistochemistry and FISH analysis of in vivo-treated *EWSR1-FLI1* and *BCR-ABL* models. a, Representative histological H&E images in A673 experimental and control xenograft tumors. n=3. Scale bars, 500 um (4×) or 50 um (20×). **b,** Representative FISH images (break-apart probe) showing t(11;22) translocation in the three PDX tumors used in the study (n=3, independent studies). Scale bars, 5 um.

Supplementary Figure 6

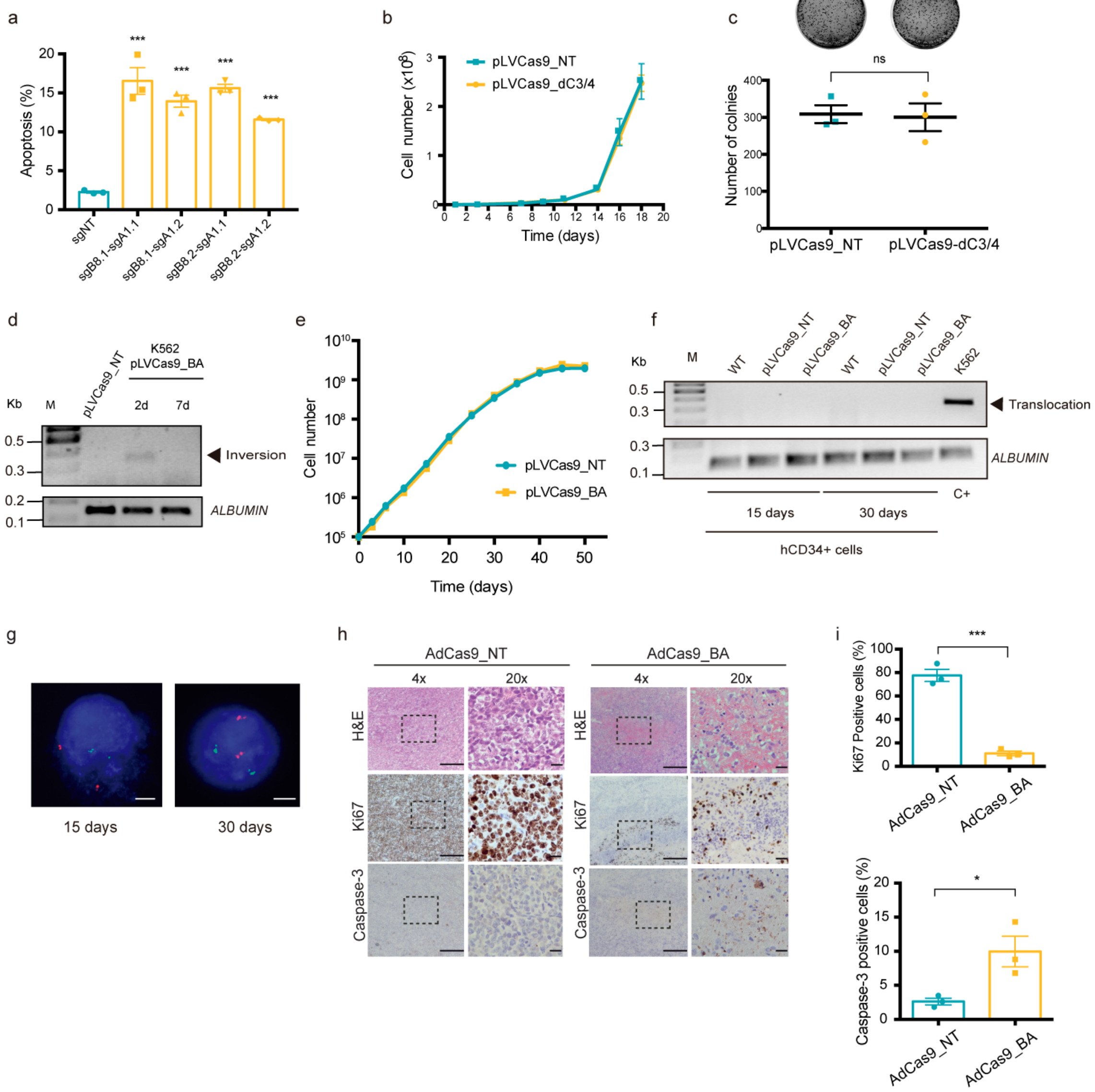

Supplementary Figure 6. **Strategy validation in CML-initiating *BCR-ABL* model. a**, DNA profile analysis by propidium iodide staining and flow cytometry. The percentage of cellular apoptosis was calculated using the percentage of the subG1 peak. The graph shows subG1 analysis. (n=3), sgNT vs sgB8.1-sgA1.1 ***p=0.001, sgNT vs sgB8.1-sgA1.2 ***p=1e-4, sgNT vs sgB8.2-sgA1.1 ***p=1e-5, sgNT vs sgB8.2-sgA1.2 ***p=7e-7. **b**, Growth rate assay curve of K562 electroporated with a non-targeting sgRNA control (LVCas9_NT) and dual control plasmid expressing two sgRNAs targeting two unrelated loci in two different chromosomes (LVCas9_dC3/4) cells (n=6). **c**, Representative crystal violet staining and statistical analysis of number of colonies of K562 electroporated with a non-targeting sgRNA control (LVCas9_NT) and dual

control plasmid expressing two sgRNAs targeting two unrelated loci in two different chromosomes (LVCas9_dC3/4) (n=3). **d,** Agarose gel electrophoresis of the *BCR-ABL1* inversion products of edited and control K562 cells (n=3, independent studies). **e,** Growth rate assay curve of CD34+ edited (LVCas9_BA) and control (LVCas9_NT) cells (n=3). **f,** PCR analysis to detect undesired translocations in wild-type cells using oligos flanking the DNA targeted by sgB8.1 and sgA1.1 on LVCas9_NT and LVCas9_BA treated CD34+ cells (n=3, independent studies). LVCas9_BA treated K562 cells are used as positive control. **g,** FISH analysis (dual color dual fusion probe) performed on LVCas9_BA treated CD34+ cells showing representative interphase nuclei at days 15 and 30 p.t. (n=3, independent studies). The green signals represent the two normal chromosomes 9 (*BCR* gene locus) and red signals the two normal chromosomes 22 (*ABL1* gene locus) Scale bars, 5 um. **h,** Representative H&E and immunostaining images of Ki-67 and caspase-3 in K562 cell experimental and control CML xenograft tumors. n=3. Scale bars, 500 um (4×) or 50 um (20×). **i,** Percentage of Ki-67-(***p=3e-4) and caspase-3 (*p=0.03) -positive cells was calculated per field analyzed. The plots show medians and ranges; error bars indicate the s.e.m. for the averages across the multiple experiments; p-values are represented (n.s. non-significant, *p≤0.05; **p≤0.01; ***p≤0.001). N corresponds with biologically independent experiments. Two-tailed unpaired *t*-test was used for statistical analysis.

Supplementary Figure 7

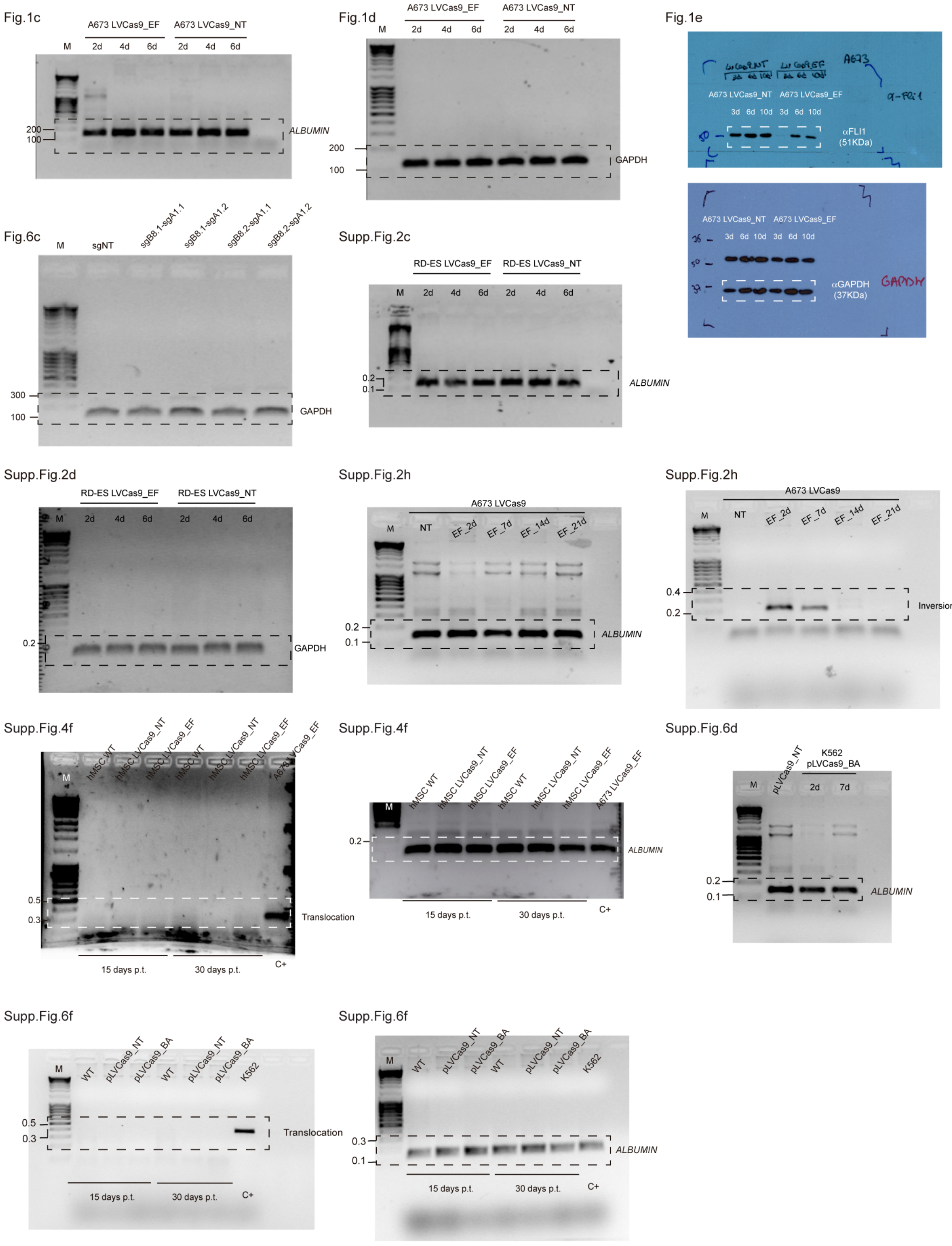

Supplementary Figure 7. Uncropped gel and immunoblot images

Supplementary Figure 8

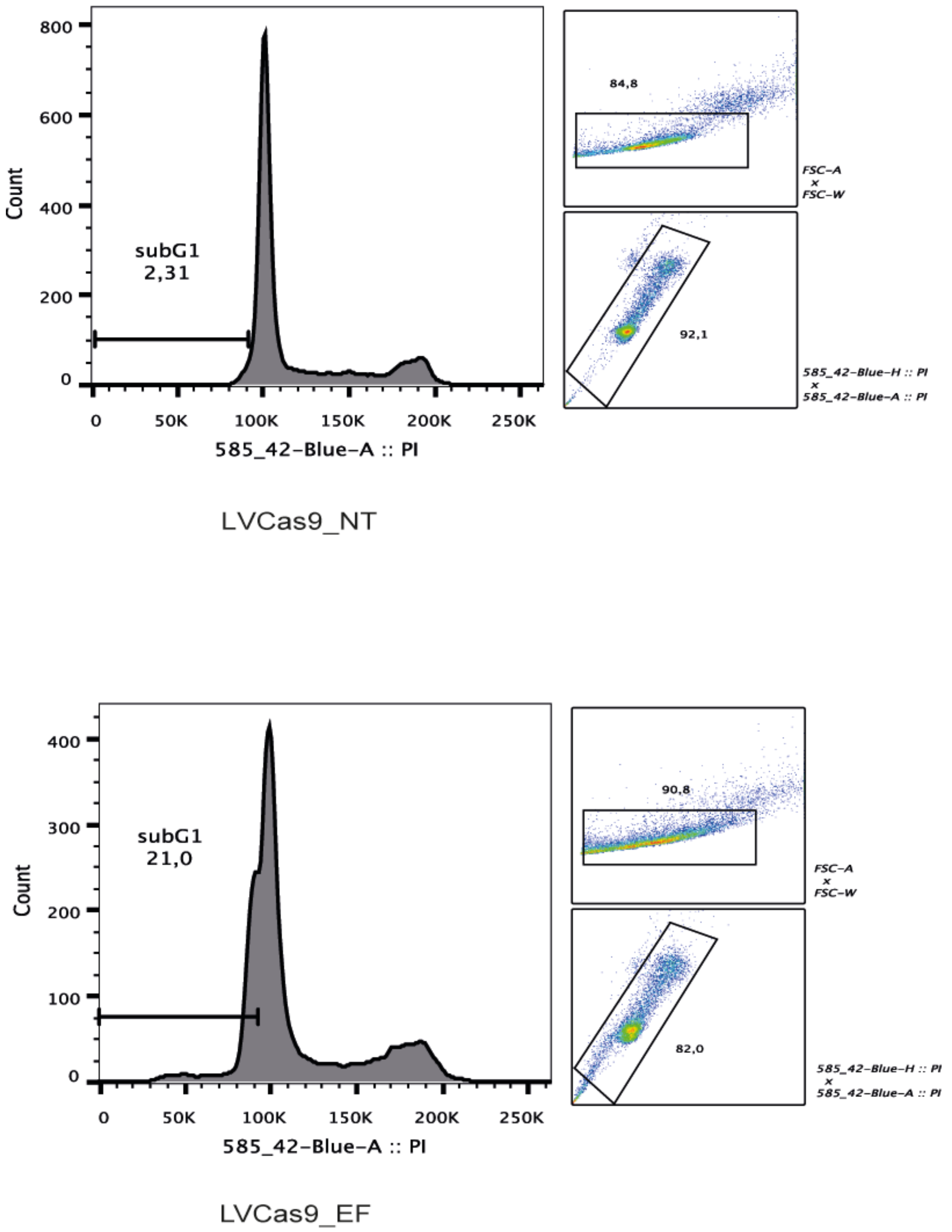

Supplementary Figure 8. Gating strategy used for SubG1 flow cytometry assay.

**sgRNAs**			
sgE3	TGGTTGCACAGTAAGTGGCG	sgF6	ATTTGGACCTGTGGCGATAT
sgE6	TAGATCGCGTACTCCATCCT	sgF8	AGTGGGCCACACTGCGACAA
sgB8.1	GACATGACCATGGGTAAGCG	sgA1.1	CACGAGGTTGACGCACCAGA
sgB8.2	TATCCGAGGCACGTTAAGGG	sgA1.2	TCCCTAATAGTGATGGCGCT
sgdC1	GACATGACCATGGGTAAGCG	sgdC3	TGGTTGCACAGTAAGTGGCG
sgdC2	CACGAGGTTGACGCACCAGA	sgdC4	AGTGGGCCACACTGCGACAA
sgNT	CCGCGCCGTTAGGGAACGAG		
**PCR/RT-PCR EF deletion detection primers**			
Ex3 *EWSR1* fw	GCCCAGCCCACTCAAGGATA	Ex9 *FLI1* rv	TTGGGGTTGGGGTAGATTCC
RT-*EWSF1I* fw	GCAGGGCTACAGTGCTTAC	RT-*EWSFLI* rv	GCAGCTCCAGGAGGAATTG
**On target detection primers**			
EWSR1 OT fw	GAGGATGATATGACCATTCTGC	*EWSR1* OT rv	CTAGGCTTTTCAACAGACTCTT
FLI1 OT fw	AACTCTCAAGAGGGTCCTTC	*FLI1* OT rv	TAATGACCAGGCACTAAGGAATG
**RT-PCR BA deletion detection primers**			
q*BCR-ABL1* fw	GATGCCAAGGATCCAACGAC	q*BCR-ABL1* rv	GGCTTCACACCATTCCCCAT
**PCR/RT-PCR/qPCR controls primers**			
Albumin fw	GCTGTCATCTCTTGTGGGCTGT	*Albumin* rv	ACTCATGGGAGCTGCTGGTTC
GAPDH fw	TCCAAAATCAAGTGGGGCGA	*GAPDH* rv	TGATGACCCTTTTGGCTCCC
GUSB fw	GAAGAAGTGGTGCGTAGGGA	*GUSB* rv	AAGGATTTGGTGTGAGCGAT
**qPCR gene expresion primers**			
q*EWSR1* fw	GGAGAGAAAATGGCGTCCAC	q*EWSR1* rv	GCTTTGTTGCCCATATGCCT
q*FLI1* fw	GGCTGGTCTTGAACTCCTGG	qFLI1 rv	GCAGCTCCAGGAGGAATTG
**qPCR deletion percentage**			
q*EF* fw	CCCAGCCAGATCCGTATCAG	q*EF* rv	GCAGCTCCAGGAGGAATTG
q*BA* fw	AGGAAGATGATGAGTCTCCGG	q*BA* rv	TGAGGCTCAAAGTCAGATGCT
**PCR inversions**			
qEWSR1 rv	CTAGGCTTTTCAACAGACTCTT	q*FLI1* rv	TAATGACCAGGCACTAAGGAATG
qBCR fw	GGTTGCTTCACAAAGGCAGGG	qABL fw	AATTTCCTGTGTGGGCCTGG
**PCR translocations**			
EWSR1 fw	GAGGATGATATGACCATTCTGC	*FLI1* rv	TAATGACCAGGCACTAAGGAATG
BCR fw	GGTTGCTTCACAAAGGCAGGG	ABL rv	GGCTGGTCTTGAACTCCTGG
**Deep sequencing primers**			
EWSR1 ont NGS fw	GAGGATGATATGACCATTCTGC	*EWSR1* ont NGS rv	CTAGGCTTTTCAACAGACTCTT
EWSR1 offT1 NGS fw	TCACTTACTGGTTGTTAAACAC	*EWSR1* offT1 NGS rv	TGTTTGTTGGAATAAACAGTGC
EWSR1 offT2 NGS fw	CCATATCTCAGCCATTTCAGTC	*EWSR1* offT3 NGS rv	CAGAGAAGGACATTGCCATTAAC
EWSR1 offT3 NGS fw	GTTCTCTGCACATAAACACACC	*EWSR1* offT3 NGS rv	CACTCAGCTTCTTGGACATGGTG
EWSR1 offT4 NGS fw	ACTTTTCTTTGGAAAGTTTAGGTAGC	*EWSR1* offT4 NGS rv	GAGTGTAAAAGACATTTCCTTAGGA
EWSR1 offT5 NGS fw	GTGTAGCCAACAGGTAGACAG	*EWSR1* offT5 NGS rv	TCATCGGGGCCTTTATTCAT
EWSR1 offT6 NGS fw	AAAGGCCTGTCTCCTTTCTCTTTC	*EWSR1* offT6 NGS rv	CTTGGCGCAGACTAGTTGTTC
EWSR1 offT7 NGS fw	TGCAGTCTTTCCCATTCCAGG	*EWSR1* offT7 NGS rv	GCTCTGTTTTGCTAAGGGCTT
EWSR1 offT8 NGS fw	AGCACAGAACATGAGCCTTGG	*EWSR1* offT8 NGS rv	GTTCTGTAGACTTGGCATCCC
EWSR1 offT9 NGS fw	CTGGCTGCTTCAATTTGATCTTC	*EWSR1* offT9 NGS rv	GTATGCAGGCCCATGTAGTC
EWSR1 offT10 NGS fw	GCCAGATGATATCCTGAAGAAT	*EWSR1* offT10 NGS rv	CTGTCCATAATACATATCAGACTC
EWSR1 offT11 NGS fw	GGGTGACCTTCTCTCTGAGCTG	*EWSR1* offT11 NGS rv	CACTCCAAGCTCTGGAAAGGG
EWSR1 offT12 NGS fw	AGTTCTACGATCTTATGGGC	*EWSR1* offT12 NGS rv	AGAGTATTCAGACTTCAGTGG
EWSR1 offT13 NGS fw	CCCACCTGAGTCCTTTCTCTG	*EWSR1* offT13 NGS rv	GAATCTTCACAGCCAGTTGTC
EWSR1 offT14 NGS fw	GCGACAGCCTCAATGCATTAGG	*EWSR1* offT14 NGS rv	CCTGGAAGCTGATGCTGCTG
EWSR1 offT15 NGS fw	TCTTAGAACCAGCAGATAGACAC	*EWSR1* offT15 NGS rv	TGGCAAACAGTATGGATGAATCC
EWSR1 offT16 NGS fw	CAGTGAAATGTCCCTGTCCATC	*EWSR1* offT16 NGS rv	TCTTGTCAATGCAGGAGAGGC
EWSR1 offT17 NGS fw	CCCAGCTACCACTCTCCATTC	*EWSR1* offT17 NGS rv	GATGGCAATGATGGCGATGATG
EWSR1 offT18 NGS fw	CTCCTATTGCTCATCGCTATG	*EWSR1* offT18 NGS rv	TACCCAGTTAATGCTGAGGAG
EWSR1 offT19 NGS fw	TCTGAAGGACAGTATCTAAGTTAAG	*EWSR1* offT19 NGS rv	CTAAAATATTTGCCTGAGAACCAG
EWSR1 offT20 NGS fw	ACAAGTTTGTGGTTTCTATGTTAGG	*EWSR1* offT20 NGS rv	TTTCATGAGTCAGTTATCCTGTTTTCC
EWSR1 offT21 NGS fw	TTGCTATATTCCTAGTGTACCTG	*EWSR1* offT21 NGS rv	GGGATAACAACATCTATGCTACC
FLI1 ont NGS fw	AACTCTCAAGAGGGTCCTTC	*FLI1* ont NGS rv	TAATGACCAGGCACTAAGGAATG
FLI1 offT1 NGS fw	TTTGCTGGAAACCCTGAAGTAC	*FLI1* offT1 NGS rv	CCCTCTCTTTCTGTTCCAGTG
FLI1 offT2 NGS fw	GGAAGCACTGCCATACACAG	*FLI1* offT2 NGS rv	TTCGCTTCTTTGTACTCCCTGTC
FLI1 offT3 NGS fw	GAGAGGACCGAAGCTTCC	*FLI1* offT3 NGS rv	CGGTCACACAGGGACTAAAAC
FLI1 offT4 NGS fw	TGTCACTATTGTTTATCCTCACAG	*FLI1* offT4 NGS rv	GTTGTCATTTACACTCTTTCTCC
FLI1 offT5 NGS fw	TCTGTTTCTGTGCATATCATCTCC	*FLI1* offT5 NGS rv	GAGAACCCATCCTAAACTAAGAG
FLI1 offT6 NGS fw	GAGGAGACCGATGGACAGAC	*FLI1* offT6 NGS rv	GCTTGGTAGCCCTTGGCC
FLI1 offT7 NGS fw	TGCTTCATAGTTGTTCCATAAG	*FLI1* offT7 NGS rv	CATCTCAAGCATCAAAGAAG
FLI1 offT8 NGS fw	TAAAACAGGAAGTGAGAGTCAAAG	*FLI1* offT8 NGS rv	CTTGAAGTGAGTTTCCATAGACTC
FLI1 offT9 NGS fw	CAGCTCCAGTCTCCCGTTC	*FLI1* offT9 NGS rv	GACTCGCTCACTTCCCATGG
FLI1 offT10 NGS fw	CAGCAGCAGCCATTTAGGAAG	*FLI1* offT10 NGS rv	GAAACTTTCCTGCACAGCTCTTG
FLI1 offT11 NGS fw	TGCCCAGGTATCAAAAGATG	*FLI1* offT11 NGS rv	TCTTTGTAAAAGAGACTTGACTGC
FLI1 offT12 NGS fw	GGAGGACCTGTATGTTGTGAG	*FLI1* offT12 NGS rv	AGCTCTGCCTTTAATTCTCAGG
FLI1 offT13 NGS fw	GAGTCTATACTGAAGTCCACTTAC	*FLI1* offT13 NGS rv	GCTATCAATAACTTTCTGTCATAG
FLI1 offT14 NGS fw	GTGAGAACTACAGGCCAGTTTC	*FLI1* offT14 NGS rv	CAATCACTGGAACAGAAAGAGAGGG
FLI1 offT15 NGS fw	ACAAAGGTAGCTGTGGATAGTCTA	*FLI1* offT15 NGS rv	GGTGTTTGAGAGAAGATACAGC
FLI1 offT16 NGS fw	TACTTCAGACAATATCCTAGTGAGAC	*FLI1* offT16 NGS rv	AGCCACAAACTGAAAATATCATGG
FLI1 offT17 NGS fw	CACTCCTAGCTTGAACACTATTTG	*FLI1* offT17 NGS rv	CAGTCACCATATCCAGGATAGC
FLI1 offT18 NGS fw	ACTGATCCACATGCTAGAGAGTG	*FLI1* offT18 NGS rv	ATGGATATCTGTTGCTCCATCC
FLI1 offT19 NGS fw	ATAGCTGGAATTAACCTGGACC	*FLI1* offT19 NGS rv	AAAGCTGAATTTCACTCTCCCC
FLI1 offT20 NGS fw	TCCACTTCCTCAAAGCTATAATC	*FLI1* offT20 NGS rv	TTTAGGATTTTTGCTTATGGCAGTG
FLI1 offT21 NGS fw	AGAACAGTATTTGCAAGGTGCTC	*FLI1* offT21 NGS rv	TAGTTTTGCCATGGGATGTGGTG
FLI1 offT22 NGS fw	TGATTGGCTTCTCAGTTAGCTTC	*FLI1* offT22 NGS rv	CTAGAAGCCACTTTCCTCAGAC
FLI1 offT23 NGS fw	CATTGATCAGCCTGAACCTGG	*FLI1* offT23 NGS rv	GTGAAGGCATGTCTGATTGTGG
FLI1 offT24 NGS fw	GCTTACATGTCTCCTGAAAGCAC	*FLI1* offT24 NGS rv	GACTTCTGTAGAAGTTTCCAGAG
FLI1 offT25 NGS fw	CTGGTACTCACCCTCTTCTC	*FLI1* offT25 NGS rv	CTCCCTGCATATTGATGTGAGG
FLI1 offT26 NGS fw	AGGTCTATAAAAGAATGTGCCCAC	*FLI1* offT26 NGS rv	GGTTGCTATACAAAGCTGTTGTTG
FLI1 offT27 NGS fw	TTCCTGAACAACCTCTCTAACTC	*FLI1* offT27 NGS rv	AAAAGCTACTGTGAGTGGGTCC
BCR ont NGS fw	GGTTGCTTCACAAAGGCAGGG	*BCR* ont NGS rv	AGCATCTGCCCCAGGAATGG
BCR offT1 NGS fw	GTCCCTATTTTCTGGCCCCTAT	*BCR* offT1 NGS rv	ACCCATTCGTCTTTCCACTT
BCR offT2 NGS fw	TTCCCCTCCACCGTATGACC	*BCR* offT2 NGS rv	CCAGTTGGAGAGCTATGCTGTTC
BCR offT3 NGS fw	GAGGCCATGATGGTGAGGAG	*BCR* offT3 NGS rv	ACTTGCCTCATCTACCTGGG
BCR offT4 NGS fw	CCTTTTCCTTTCGGTTGGGC	*BCR* offT4 NGS rv	CACCTTGCCCTTTCACTGTTC
BCR offT5 NGS fw	AGCACAGACAAGTCCCATGC	*BCR* offT5 NGS rv	CCAAGCAGGAGGTGACAAATG
ABL1 ont NGS fw	AATTTCCTGTGTGGGCCTGG	*ABL1* ont NGS rv	GGCTGGTCTTGAACTCCTGG
ABL1 offT1 NGS fw	ACAATTTGATTCCCACCCCA	*ABL1* offT1 NGS rv	GCATTCACAGTACCCATGCC
ABL1 offT2 NGS fw	CTCAACCACTTCCTCTCTCCTC	*ABL1* offT2 NGS rv	CATGGCCCCTGTTGAAGAAG
ABL1 offT3 NGS fw	CGAATGAATGTGTGGCAGGC	*ABL1* offT3 NGS rv	GGACAAGGGGTTGCTGAGG
ABL1 offT4 NGS fw	GCAGATCCCCACAGTCAGAA	*ABL1* offT4 NGS rv	ATGGCCCAGTGAGTAGGTCC
ABL1 offT5 NGS fw	TGCAAAGACGGCTAGGAAGG	*ABL1* offT5 NGS rv	TCGACACGTACCCTCACAG

Supplementary Table 1. sgRNA sequences used for CRISPR-based gene editing. Oligo sequences used for PCR, RT-PCR and NGS analysis.

a

				Edited		
Guide	sgRNA Sequences (5'-3')	Chromosome	Position	Total reads	Modified Reads	Editing efficiency (%)
sgE3	TGGTTGCACAGTAAGTGGCG	22	29272832	22981	14207	61.8

Sequence	Reads
GCAGTGCATAGATATTAAGTAACTTGCCAGTGGTTGCACAGTAAGTGGCGGGGTTAGCTCTAAAAACTGGCGACCTAGCCAA	x8778
GCAGTGCATAGATATTAAGTAACTTGCCAGTGGTTGCACAGTAAGT-GCGGGGTTAGCTCTAAAAACTGGCGACCTAGCCAA	x1459
GCAGTGCATAGATATTAAGTAACTTGCCAGTGGTTGCACAGTAAGTGTGGCGGGGTTAGCTCTAAAAACTGGCGACCTAGCCAA	x835
GCAGTGCATAGATATTAAGTAACTTGCCAGTGGTTGCACAGTAAGTGGGCGGGGTTAGCTCTAAAAACTGGCGACCTAGCCAA	x763
GCAGTGCATAGATATTAAGTAACTTGCCAGTGGTTGCACAGTAA---GCGGGGTTAGCTCTAAAAACTGGCGACCTAGCCAA	x688
GCAGTGCATAGATATTAAGTAACTTGCCAGTGGTT------------GCGGGGTTAGCTCTAAAAACTGGCGACCTAGCCAA	x526
GCAGTGCATAGATATTAAGTAACTTGCCAGTGGTTGCACAGTAA---GGCGGGGTTAGCTCTAAAAACTGGCGACCTAGCCAA	x446
GCAGTGCATAGATATTAAGTAACTTGCC---------------AGTGGCGGGGTTAGCTCTAAAAACTGGCGACCTAGCCAA	x343
GCAGTGCATAGATATTAAGTAACTTGCCAGTGGTTGCACA-------GCGGGGTTAGCTCTAAAAACTGGCGACCTAGCCAA	x286
GCAGTGCATAGATATTAAGTAACTTGCCAGTGGTTGCACA------------GTTAGCTCTAAAAACTGGCGACCTAGCCAA	x254
GCAGTGCATAGATATTAAGTAACTTGCCAGT--------------------GGTTAGCTCTAAAAACTGGCGACCTAGCCAA	x218
GCAGTGCATAGATATTAAGTAACTTGCCAGTGGTTGCACAGTA----GCGGGGTTAGCTCTAAAAACTGGCGACCTAGCCAA	x213
…	

b

				Edited		
Guide	sgRNA Sequences (5'-3')	Chromosome	Position	Total reads	Modified Reads	Editing efficiency (%)
sgF8	AGTGGGCCACACTGCGACAA	11	128809772	12315	8151	66.2

Sequence	Reads
TGGGTAGGCAGAGTCCCTGGGATGGGAAGGTGAGTGGGCCACACTGCGACAAGGGCCTGCTAGCTCCCAATCTCGATGGACTT	x4162
TGGGTAGGCAGAGTCCCTGGGATGGGAAGGTGAGTGGGCCACACTGCGAACAAGGGCCTGCTAGCTCCCAATCTCGATGGACTT	x1111
TGGGTAGGCAGAGTCCCTGGGATGGGAAGGTGAGTGGGCCACACTGC-------------TAGCTCCCAATCTCGATGGACTT	x622
TGGGTAGGCAGAGTCCCTGGGATGGGAAGGTGAGTGGGCC-----------------TGCTAGCTCCCAATCTCGATGGACTT	x515
TGGGTAGGCAGAGTCCCTGGGATGGGAAGGTGAGTGGGCCACACTGCGA-AAGGGCCTGCTAGCTCCCAATCTCGATGGACTT	x502
TGGGTAGGCAGAGTCCCTGGGATGGGAAGGTGAGTGGGCCACACTGCGA---GGGCCTGCTAGCTCCCAATCTCGATGGACTT	x411
TGGGTAGGCAGAGTCCCTGGGATGGGAAGGTGAGTGGGCCACACTGC---AAGGGCCTGCTAGCTCCCAATCTCGATGGACTT	x326
TGGGTAGGCAGAGTCCCTGGGATGGGAAGGTGAGTGGGCCACACTGCGA--AGGGCCTGCTAGCTCCCAATCTCGATGGACTT	x197
TGGGTAGGCAGAGTCCCTGGGATGGGAAGGTGAGTGGGCCACACTGCGA---------GCTAGCTCCCAATCTCGATGGACTT	x191
TGGGTAGGCAGAGTCCCTGGGATGGGAAGGTGAGTGGGCCACACTG-----------------CTCCCAATCTCGATGGACTT	x173
TGGGTAGGCAGAGTCCCTGGGATGGGAAGGTGAGTGGGCCACACTGCG-CAAGGGCCTGCTAGCTCCCAATCTCGATGGACTT	x155
TGGGTAGGCAGAGTCCCTGGGATGGGAAGGTGAGTGGGCCACA--------AGGGCCTGCTAGCTCCCAATCTCGATGGACTT	x147
…	

c

				Edited		
Guide	sgRNA Sequences (5'-3')	Chromosome	Position	Total reads	Modified Reads	Editing efficiency (%)
sgB8.2	TATCCGAGGCACGTTAAGGG	22	23276627	126845	108246	85.3

Sequence	Reads
CAGCACCCCTTCACTCAGAGCTTTCTATCCGAGGCACGTTAAGGGAGGTGGCTTGATGGTGGCTAAGGCAGGATGATAGATG	x18646
CAGCACCCCTTCACTCAGAGCTTTCTATCCGAGGCACGTTGAAGGGAGGTGGCTTGATGGTGGCTAAGGCAGGATGATAGATG	x69906
CAGCACCCCTTCACTCAGAGCTTTCTATCCGAGGCACGTTAAAAGGGAGGTGGCTTGATGGTGGCTAAGGCAGGATGATAGATG	x5692
CAGCACCCCTTCACTCAGAGCTTTCTATCCGAGGCACGTT-AGGGAGGTGGCTTGATGGTGGCTAAGGCAGGATGATAGATG	x5585
CAGCACCCCTTCACTCAGAGCTTTCTATACGAGGCACGTTAAAGGGAGGTGGCTTGATGGTGGCTAAGGCAGGATGATAGATG	x2758
CAGCACCCCTTCACTCAGAGCTTTCTATCCG----------AGGGAGGTGGCTTGATGGTGGCTAAGGCAGGATGATAGATG	x1922
CAGCACCCCTTCACTCAGAGCTTTCTATCCGAGGCACGTTAA-GGAGGTGGCTTGATGGTGGCTAAGGCAGGATGATAGATG	x955
CAGCACCCCTTCACTCAGAGCTTTCTATCCGAGGCACGTTAA-----GTGGCTTGATGGTGGCTAAGGCAGGATGATAGATG	x792
CAGCACCCCTTCACTCAGAGCTTTCTATCCGAGGCACGTT--GGGAGGTGGCTTGATGGTGGCTAAGGCAGGATGATAGATG	x631
CAGCACCCCTTCACTCAGAGCTTTCTATCCGAGGCACGTT--GGGAGGTGGCTTGATGGTGGCTAAGGCAGGATGATAGATG	x618
CAGCACCCCTTCACTCAGAGCTTTCTATCC--------------GAGGTGGCTTGATGGTGGCTAAGGCAGGATGATAGATG	x613
CAGCACCCCTTCACTCAGAGCTTTCTATCCGA-----------------GGCTTGATGGTGGCTAAGGCAGGATGATAGATG	x548
…	

d

				Edited		
Guide	sgRNA Sequences (5'-3')	Chromosome	Position	Total reads	Modified Reads	Editing efficiency (%)
sgA1.1	CACGAGGTTGACGCACCAGA	9	130848122	68241	55669	81.6

Sequence	Reads
TTGTATGCTCATGGCCACGAGGTTGACGCACCAGAGGGAGGCACTCAAGTGCCTTTTCAGGACAGCCTGAAGGAAGCCTCCC	x12556
TTGTATGCTCATGGCCACGAGGTTGACGCACCCAGAGGGAGGCACTCAAGTGCCTTTTCAGGACAGCCTGAAGGAAGCCTCCC	x19018
TTGTATGCTCATGGCCACGAGGTTGACGCA-CAGAGGGAGGCACTCAAGTGCCTTTTCAGGACAGCCTGAAGGAAGCCTCCC	x18028
TTGTATGCTCATGGCCACGAGGTTGACGCACC-GAGGGAGGCACTCAAGTGCCTTTTCAGGACAGCCTGAAGGAAGCCTCCC	x4541
TTGTATGCTCATGGCCACGAGGTTGACG---CAGAGGGAGGCACTCAAGTGCCTTTTCAGGACAGCCTGAAGGAAGCCTCCC	x3842
TTGTATGCTCATGGCCACGAGGTTGACGCACCCAGAGGGAGGCACTCAAGTGCCTTTTCAGGACAGCCTGAAGGAAGCCTCCC	x1105
TTGTATGCTCATGGCCACGAGGTTGACGCA-CAGAGGGAGGCACTCAAGTGCCTTTTCAGGACAGCCTGAAGGAAGCCTCCC	x1036
TTGTATGCTCATGGCCACGAGGTTGAC-------------GCACTCAAGTGCCTTTTCAGGACAGCCTGAAGGAAGCCTCCC	x927
TTGTATGCTCATGGCCACGAGGTTGACG--CCAGAGGGAGGCACTCAAGTGCCTTTTCAGGACAGCCTGAAGGAAGCCTCCC	x436
TTGTATGCTCATGGCCACGAGGTTGACGCACC-GAGGGAGGCACTCAAGTGCCTTTTCAGGACAGCCTGAAGGAAGCCTCCC	x289
TTGTATGCTCATGGCCACGAGGTTGACGCAC------------CTCAAGTGCCTTTTCAGGACAGCCTGAAGGAAGCCTCCC	x240
TTGTATGCTCATGGCCACGAGGTTGACG---CAGAGGGAGGCACTCAAGTGCCTTTTCAGGACAGCCTGAAGGAAGCCTCCC	x221
…	

e

Guide	sgRNA Sequences (5'-3')	Chromosome	Position	Edited		
				Total reads	Modified Reads	Editing efficiency (%)
sgE3	TGGTTGCACAGTAAGTGGCG	22	29272832	54386	7091	13

Sequence	Reads
GCAGTGCATAGATATTAAGTAACTTGCCAGTGGTTGCACAGTAAGTGGCGGGGTTAGCTCTAAAAACTGGCGACCTAGCCAA	X47295
GCAGTGCATAGATATTAAGTAACTTGCCAGTGGTTGCACAGTAAGT-GCGGGGTTAGCTCTAAAAACTGGCGACCTAGCCAA	X648
GCAGTGCATAGATATTAAGTAACTTGCCAGTGGTTGCACAGTAAGTGTGGCGGGGTTAGCTCTAAAAACTGGCGACCTAGCCAA	X529
GCAGTGCATAGATATTAAGTA---ACCTAGCCAA	X427
G--GCGGGGTTAGCTCTAAAAACTGGCGACCTAGCCAA	X338
G--GCCAA	X324
--	X214
GCAGTGCATAGATATTAAGTAACTTGCCAGTGGTTGCACAGTAAGTGGGCGGGGTTAGCTCTAAAAACTGGCGACCTAGCCAA	X177
GCAGTGCATAGATATTAAGTAACTTGCCAGTGGTTGCACAGTAA---GCGGGGTTAGCTCTAAAAACTGGCGACCTAGCCAA	X168
GCAGTGCATAGATATTAAGTAACTTGCCAGTGGTT---------------------GCTCTAAAAACTGGCGACCTAGCCAA	X134
GCAGTGCATAGATATTAAGTAACTTGCCAGTGGTTGCACAGTA--TGGCGGGGTTAGCTCTAAAAACTGGCGACCTAGCCAA	X126
GCAGTGCATAGATATTAAGTAACTTGCCAGT--------------------------------AAACTGGCGACCTAGCCAA	X110
…	

Guide	sgRNA Sequences (5'-3')	Chromosome	Position	Edited		
				Total reads	Modified Reads	Editing efficiency (%)
sgF8	AGTGGGCCACACTGCGACAA	11	128809772	1057	34	3.2

Sequence	Reads
TGGGTAGGCAGAGTCCCTGGGATGGGAAGGTGAGTGGGCCACACTGCGACAAGGGCCTGCTAGCTCCCAATCTCGATGGACTT	X1023
TGGGTAGGCAGAGTCCCTGGGATGGGAAGGTGAGTGGGCCACACT-----------------GCTCCCAATCTCGATGGACTT	X5
TGGGTAGGCAGAGTCCCTGGGATGGGAAGGTGAGTGGGCCACACTGCG------------TAGCTCCCAATCTCGATGGACTT	X4
TGGGTAGGCAGAGTCCCTGGGATGGGAAGGTGAGTGGGCCACACTGCGAACAAGGGCCTGCTAGCTCCCAATCTCGATGGACTT	X4
TGGGTAGGCAGAGTCCCTGGGATGGGAAGGTGAGTGGGCC-----------------TGCTAGCTCCCAATCTCGATGGACTT	X3
TGGGTAGGCAGAGTCCCTGGGATGGGAAGGTGAGTGG-CCACTAGCTCCCAAGGAAGGGCCTGCTAGCTCCCAATCTCGATGGACTT	X3
TGGGTAGGCAGAGTCCCTGGGATGGGAAGGTGAGTGGGCCACACTGCG-CAAGGGCCTGCTAGCTCCCAATCTCGATGGACTT	X3
TGGGTAGGCAGAGTCCCTGGGATGGGAAGGTGAGTGGGCCACAC------AAGGGCCTGCTAGCTCCCAATCTCGATGGACTT	X2
TGGGTAGGCAGAGTCCCTGGGATGGGAAGGTGAGTGGGCC-----------------TGCTAGCTCCCAATCTCGATGGACTT	X2
TGGGTAGGCAGAGTCCCTGGGATGGGAAGGTGAGTG---	X2
TGGGTAGGCAGAGTCCCTGGGATGGGAAGGTG--	X2
TGGGTAGGCAGAGTCCCTGGGATGGGAAGGTGAGTGGGCCACACTGCGA-AAGGGCCTGCTAGCTCCCAATCTCGATGGACTT	X2
…	

f

Guide	sgRNA Sequences (5'-3')	Chromosome	Position	Edited		
				Total reads	Modified Reads	Editing efficiency (%)
sgE3	TGGTTGCACAGTAAGTGGCG	22	29272832	19824	2036	10.3

Sequence	Reads
GCAGTGCATAGATATTAAGTAACTTGCCAGTGGTTGCACAGTAAGTGGCGGGGTTAGCTCTAAAAACTGGCGACCTAGCCAA	x17788
GCAGTGCATAGATATTAAGTAACTTGCCAGTGGTTGCACAGTAAGT-GCGGGGTTAGCTCTAAAAACTGGCGACCTAGCCAA	x251
GCAGTGCATAGATATTAAGTAACTTGCCAGTGGTTGCACAGTAAGTGGGCGGGGTTAGCTCTAAAAACTGGCGACCTAGCCAA	X172
GCAGTGCATAGATATTAAGTAACTTGCCAGTGGTTGCACAGTAAGTGTGGCGGGGTTAGCTCTAAAAACTGGCGACCTAGCCAA	X162
GCAGTGCATAGATATTAAGTAACTTGCCAGTGGTTGCACAGTAA---GCGGGGTTAGCTCTAAAAACTGGCGACCTAGCCAA	X155
GCAGTGCATAGATATTAAGTAACTTGCCAGTGGTTGCACAGTAA--GGCGGGGTTAGCTCTAAAAACTGGCGACCTAGCCAA	X66
GCAGTGCATAGATATTAAGTAACTTGCCAGTGGTT------------GCGGGGTTAGCTCTAAAAACTGGCGACCTAGCCAA	X54
GCAGTGCATAGATATTAAGTAACTTGCCA------------------GCGGGGTTAGCTCTAAAAACTGGCGACCTAGCCAA	X42
GCAGTGCATAGATATTAAGTAACTTGCCAGTGGTTGCACAGTA----GCGGGGTTAGCTCTAAAAACTGGCGACCTAGCCAA	X37
GCAGTGCATAGATATTAAGTAACTTGCCAGTGGTTGCACA------------GTTAGCTCTAAAAACTGGCGACCTAGCCAA	X36
GCAGTGCATAGATATTAAGTAACTTGCCAGTGGTTGCACAGTAA--------GTTAGCTCTAAAAACTGGCGACCTAGCCAA	X32
GCAGTGCATAGATATTAAGTAACTTGCCAGTGGTTGCACA-------GCGGGGTTAGCTCTAAAAACTGGCGACCTAGCCAA	X30
…	

Guide	sgRNA Sequences (5'-3')	Chromosome	Position	Edited		
				Total reads	Modified Reads	Editing efficiency (%)
sgF8	AGTGGGCCACACTGCGACAA	11	128809772	12336	425	3.4

Sequence	Reads
TGGGTAGGCAGAGTCCCTGGGATGGGAAGGTGAGTGGGCCACACTGCGACAAGGGCCTGCTAGCTCCCAATCTCGATGGACTT	X11911
TGGGTAGGCAGAGTCCCTGGGATGGGAAGGTGAGTGGGCCACACTGCGAACAAGGGCCTGCTAGCTCCCAATCTCGATGGACTT	X54
TGGGTAGGCAGAGTCCCTGGGATGGGAAGGTGAGTGGGCC-----------------TGCTAGCTCCCAATCTCGATGGACTT	X35
TGGGTAGGCAGAGTCCCTGGGATGGGAAGGTGAGTGGGCCACACTGC---AAGGGCCTGCTAGCTCCCAATCTCGATGGACTT	X27
TGGGTAGGCAGAGTCCCTGGGATGGGAAGGTGAGTGGGCCACACTGCGA-AAGGGCCTGCTAGCTCCCAATCTCGATGGACTT	X20
TGGGTAGGCAGAGTCCCTGGGATGGGAAGGTGAGTGGGCCACACTGC--CAAGGGCCTGCTAGCTCCCAATCTCGATGGACTT	X20
TGGGTAGGCAGAGTCCCTGGGATGGGAAGGTGAGTGGGCCACACTGCGA---GGGCCTGCTAGCTCCCAATCTCGATGGACTT	X16
TGGGTAGGCAGAGTCCCTGGGATGGGAAGGTGAGTGGGCCACACTGCGACCAAGGGCCTGCTAGCTCCCAATCTCGATGGACTT	X14
TGGGTAGGCAGAGTCCCTGGGATGGGAAGGTGAGTGGGCCACACTGCGAC----------TAGCTCCCAATCTCGATGGACTT	X13
TGGGTAGGCAGAGTCCCTGGGATGGGAAGGTGAGTGGGCCACAC------AAGGGCCTGCTAGCTCCCAATCTCGATGGACTT	X13
TGGGTAGGCAGAGTCCCTGGGATGGGAAGGTGAGTGGGCCACACTGC-------------TAGCTCCCAATCTCGATGGACTT	X11
TGGGTAGGCAGAGTCCCTGGGATGGGAAGGTGAGTGGGCCA----------AGGGCCTGCTAGCTCCCAATCTCGATGGACTT	X11
…	

g

Guide	sgRNA Sequences (5'-3')	Chromosome	Position	Edited<br>Total reads	Edited<br>Modified Reads	Edited<br>Edited cells (%)
sgB8.2	TATCCGAGGCACGTTAAGGG	22	23276627	11743	491	4.2

Sequence	Reads
CAGCACCCCTTCACTCAGAGCTTTCTATCCGAGGCACGTTAAGGGAGGTGGCTTGATGGTGGCTAAGGCAGGATGATAGATG	x11252
CAGCACCCCTTCACTCAGAGCTTTCTATCCGAGGCACGTTAAAGGGAGGTGGCTTGATGGTGGCTAAGGCAGGATGATAGATG	x295
CAGCACCCCTTCACTCAGAGCTTTCTATCCG----------AGGGAGGTGGCTTGATGGTGGCTAAGGCAGGATGATAGATG	X19
CAGCACCCCTTCACTCAGAGCTTTCTATCCG------------------GGCTTGATGGTGGCTAAGGCAGGATGATAGATG	X13
CAGCACCCCTTCACTCAGAGCTTTCTATCCGAGGCACGTTA-GGGAGGTGGCTTGATGGTGGCTAAGGCAGGATGATAGATG	X13
CAGCACCCCTTCACTCAGAGCTTTCTATCC--------------GAGGTGGCTTGATGGTGGCTAAGGCAGGATGATAGATG	X9
CAGCACCCCTTCACTCAGAGCTTTCTATCCGAGGCACG--------------TTGATGGTGGCTAAGGCAGGATGATAGATG	X9
CAGCACCCCTTCACTCAGAGCTTTCTATCCGA-----------GGAGGTGGCTTGATGGTGGCTAAGGCAGGATGATAGATG	X8
CAGCACCCCTTCACTCAGAGCTTTCTATCCGAGGCACGT-------------TTGATGGTGGCTAAGGCAGGATGATAGATG	X7
CAGCACCCCTTCACTCAGAGCTTTCT---------------------GTGGCTTGATGGTGGCTAAGGCAGGATGATAGATG	X6
CAGCACCCCTTCACTCAGAGCTTTCTATCCGAGGCACG---AGGGAGGTGGCTTGATGGTGGCTAAGGCAGGATGATAGATG	X5
CAGCACCCCTTCACTCAGAGCTTTCTATCCGAGGCACGTT---------------ATGGTGGCTAAGGCAGGATGATAGATG	x5

…

Guide	sgRNA Sequences (5'-3')	Chromosome	Position	Edited<br>Total reads	Edited<br>Modified Reads	Edited<br>Edited cells (%)
sgA1.1	CACGAGGTTGACGCACCAGA	9	130848122	9002	421	4.7

Sequence		Reads
TTGTATGCTCATGGCCACGAGGTTGACGCACCAGAGGGAGGCACTCAAGTGCCTTTTCAGGACAGCCTGAAGGAAGCCTCCC		x8581
TTGTATGCTCATGGCCACGAGGTTGACGCACCCAGAGGGAGGCACTCAAGTGCCTTTTCAGGACAGCCTGAAGGAAGCCTCCC		X198
TTGTATGCTCATGGCCACGAGGTTGAC-------------GCACTCAAGTGCCTTTTCAGGACAGCCTGAAGGAAGCCTCCC	X5	X53
TTGTATGCTCATGGCCACGAGGTTGACGCAC-AGAGGGAGGCACTCAAGTGCCTTTTCAGGACAGCCTGAAGGAAGCCTCCC		X51
TTGTATGCTCATGGCCACGAGGTTGACGCACC-GAGGGAGGCACTCAAGTGCCTTTTCAGGACAGCCTGAAGGAAGCCTCCC		X17
TTGTATGCTCATGGCCACGAGGTTGACG---CAGAGGGAGGCACTCAAGTGCCTTTTCAGGACAGCCTGAAGGAAGCCTCCC		X7
TTGTATGCTCATGGCCACGAGGTTGACGCAC------------CTCAAGTGCCTTTTCAGGACAGCCTGAAGGAAGCCTCCC		X5
TTGTATGCTCATGGCCACG-------------AGAGGGAGGCACTCAAGTGCCTTTTCAGGACAGCCTGAAGGAAGCCTCCC		X5
TTGTATGCTCATGGCCACG--------------GAGGGAGGCACTCAAGTGCCTTTTCAGGACAGCCTGAAGGAAGCCTCCC		X5
TTGTATGCTCATGGCCACGAGGTTGACGCAC--------------CAAGTGCCTTTTCAGGACAGCCTGAAGGAAGCCTCCC		X4
TTGTATGCTCATGGCCACG----------------------------AGTGCCTTTTCAGGACAGCCTGAAGGAAGCCTCCC		X4
TTGTATGCTCATGGCCACGAGGTTGACGCACC------AGGCACTCAAGTGCCTTTTCAGGACAGCCTGAAGGAAGCCTCCC		X4

…

h

Guide	sgRNA Sequences (5'-3')	Chromosome	Position	Edited<br>Total reads	Edited<br>Modified Reads	Edited<br>Edited cells (%)
sgB8.2	TATCCGAGGCACGTTAAGGG	22	23276627	11101	195	1.8

Sequence	Reads
CAGCACCCCTTCACTCAGAGCTTTCTATCCGAGGCACGTTAAGGGAGGTGGCTTGATGGTGGCTAAGGCAGGATGATAGATG	X10906
CAGCACCCCTTCACTCAGAGCTTTCTATCCGAGGCACGTTAAAGGGAGGTGGCTTGATGGTGGCTAAGGCAGGATGATAGATG	X115
CAGCACCCCTTCACTCAGAGCTTTCTATCCG----------AGGGAGGTGGCTTGATGGTGGCTAAGGCAGGATGATAGATG	X17
CAGCACCCCTTCACTCAGAGCTTTCTATCC--------------GAGGTGGCTTGATGGTGGCTAAGGCAGGATGATAGATG	X13
CAGCACCCCTTCACTCAGAGCTTTCTATCCGA-----------GGAGGTGGCTTGATGGTGGCTAAGGCAGGATGATAGATG	X10
CAGCACCCCTTCACTCAGAGCTTTCTATCCGA-----------------GGCTTGATGGTGGCTAAGGCAGGATGATAGATG	X7
CAGCACCCCTTCACTCAGAGCTTTCTATCCGAGGCAC----------GTGGCTTGATGGTGGCTAAGGCAGGATGATAGATG	X4
CAGCACCCCTTCACTCAGAGCTTTCTATCCGAGGCACGTTAA-GGAGGTGGCTTGATGGTGGCTAAGGCAGGATGATAGATG	X3
CAGCACCCCTTCACTCAGAGCTTTCTAT-----------------------CTTGATGGTGGCTAAGGCAGGATGATAGATG	X3
CAGCACCCCTTCACTCAGAGCTTTCTA--------------------GTGGCTTGATGGTGGCTAAGGCAGGATGATAGATG	X3
CAGCACCCCTTCACTCAGAGCTTTCTATC----------------------CTTGATGGTGGCTAAGGCAGGATGATAGATG	X2
CAGCACCCCTTCACTCAGAGCTTTCTATCCGAGGCACGTTAAAAGGGAGGTGGCTTGATGGTGGCTAAGGCAGGATGATAGATG	X2

…

Guide	sgRNA Sequences (5'-3')	Chromosome	Position	Edited<br>Total reads	Edited<br>Modified Reads	Edited<br>Edited cells (%)
sgA1.1	CACGAGGTTGACGCACCAGA	9	130848122	9407	130	1,4

Sequence	Reads
TTGTATGCTCATGGCCACGAGGTTGACGCACCAGAGGGAGGCACTCAAGTGCCTTTTCAGGACAGCCTGAAGGAAGCCTCCC	X9277
TTGTATGCTCATGGCCACGAGGTTGACGCACCCAGAGGGAGGCACTCAAGTGCCTTTTCAGGACAGCCTGAAGGAAGCCTCCC	X64
TTGTATGCTCATGGCCACGAGGTTGACGCAC-AGAGGGAGGCACTCAAGTGCCTTTTCAGGACAGCCTGAAGGAAGCCTCCC	X21
TTGTATGCTCATGGCCACGAGGTTGAC-------------GCACTCAAGTGCCTTTTCAGGACAGCCTGAAGGAAGCCTCCC	X16
TTGTATGCTCATGGCCACGAGGTTGACGCACC-GAGGGAGGCACTCAAGTGCCTTTTCAGGACAGCCTGAAGGAAGCCTCCC	X4
TTGTATGCTCATGGCCACGAGGTTGACGCAC----------CACTCAAGTGCCTTTTCAGGACAGCCTGAAGGAAGCCTCCC	X4
TTGTATGCTCATGGCCAC---------------GAGGGAGGCACTCAAGTGCCTTTTCAGGACAGCCTGAAGGAAGCCTCCC	X3
TTGTATGCTCATGGCCACGAGGTTGACG---CAGAGGGAGGCACTCAAGTGCCTTTTCAGGACAGCCTGAAGGAAGCCTCCC	X3
TTGTATGCTCATGGCCACGAGGTTGACGCA---------------------CCTTTTCAGGACAGCCTGAAGGAAGCCTCCC	X3
TTGTATGCTCATGGCCACGAGG----------------------TCAAGTGCCTTTTCAGGACAGCCTGAAGGAAGCCTCCC	X2
TTGTATGCTCATGGCCACGAGGT--------------------------TGCCTTTTCAGGACAGCCTGAAGGAAGCCTCCC	X2
TTGTATGCTCATGGCCACGAGGTTGACGCAC--GAGGGAGGCACTCAAGTGCCTTTTCAGGACAGCCTGAAGGAAGCCTCCC	X2

…

Supplementary Table 2. NGS analysis of the on-target *EWSR1*, *FLI1*, *BCR* and *ABL1* sites. Summary of the loci analysis (sgRNA sequence, chromosome, position, total and modified reads and editing efficiency). **a**, **b**, Indels at *EWSR1* and *FLI1* loci in A673 edited cells. **c**, **d**, Most representative indels at *BCR* and *ABL1* loci in K562 edited cells. **e**, **f**, Indels at *EWSR1* and *FLI1* loci in Ewing sarcoma tumors. **g**, **h**, Indels at *BCR* and *ABL1* loci in CML tumors. sgRNA sequence is underlined. Identified mutations are shown in red. −, deletion.

				hMSC LVCas9_NT			hMSC LVCas9_EF		
OFF Target **hMSC**	Sequences (5'-3')	Chromosome	Position	Total reads	Modified Reads	Off-target (%)	Total reads	Modified Reads	Off-target (%)
sgE3	TGGTTGCACAGTAAGTGGCG								
OFT#1	TGATTGCACTGTAAGTGGCC	chr5	78568232	49650	2	0,00	23200	5	0,02
OFT#2	TAGGTGCTCAGTAAGTGGCT	chr10	35792862	21646	0	0,00	21646	1	0,00
OFT#3	CGACTTCACAGTAAGTGGCG	chr18	74446599	39768	0	0,00	39768	0	0,00
OFT#4	AGGTAGCGCAGTTAGTGGCG	chr9	14347518	47792	0	0,00	31603	0	0,00
OFT#5	AGGTAGAACAGTAAGTGGCA	chr1	194593114	3010	0	0,00	27290	3	0,01
OFT#6	TAGTTGTTCAGTAAGTGGCA	chr1	11805340	47295	5	0,01	47691	3	0,01
OFT#7	GGGTAGCAGAGGAAGTGGCG	chr19	32779534	2985	0	0,00	40221	3	0,01
OFT#8	TGGATGCTGAGTAAGTGGCC	chrX	115433334	550	0	0,00	37235	3	0,01
OFT#9	TGCTTGCAAGGTAAGTGGCC	chr2	162011934	2487	0	0,00	36328	6	0,02
OFT#10	TGGTGGCACAGTAACTGGCC	chr4	122234874	6856	2	0,03	7208	0	0,00
OFT#11	AGGGTGCAAAGTGAGTGGCG	chr5	178332117	1302	0	0,00	1705	1	0,06
OFT#12	TGGTGGCCCAGGAAGTGGCC	chr11	15652849	2409	0	0,00	3098	0	0,00
OFT#13	AAGTTGCACAGTCAGTGGCA	chr12	117053997	2083	0	0,00	1663	0	0,00
OFT#14	GGGGTGCACAGCAAGTGGTG	chr3	172710847	2335	0	0,00	2551	0	0,00
OFT#15	AGATTGCACGGTAAGGGGCG	chr21	45185099	729	0	0,00	387	0	0,00
OFT#16	TGGCTGGACAGTCAGTGGCT	chr4	27092448	15750	0	0,00	11309	0	0,00
OFT#17	TGGCTGCAGAGGAAGTGGGG	chr12	120653158	6958	5	0,07	7520	4	0,05
OFT#18	TGTTTGCACAAGAAGTGGTG	chr3	1052161	9349	0	0,00	8813	0	0,00
OFT#19	TTATTGCACAGTAGGTGGCA	chr5	84015337	7703	0	0,00	8166	0	0,00
OFT#20	TGCTTGCATAGTAAATGGCA	chrX	20173722	2951	0	0,00	3094	0	0,00
OFT#21	AGGTTGCATAGTAAGTGTCA	chr11	74340172	5620	0	0,00	4374	0	0,00
sgF8	AGTGGGCCACACTGCGACAA								
OFT#1	GATTGGCCACACTGTGACAA	chr9	124911364	30160	8	0,03	29771	10	0,03
OFT#2	GGCGGGGCACACAGCGACAA	chr1	20716449	57401	2	0,00	23356	0	0,00
OFT#3	AGTGAGGAACACTGCGGCAA	chr6	30452057	988	0	0,00	25067	0	0,00
OFT#4	TGTGGGCCAGGCTGCGGCAA	chr14	58819512	480	0	0,00	31988	2	0,01
OFT#5	AGTGGGCTAGCCTGCGACAG	chr10	3167786	1157	0	0,00	28473	2	0,01
OFT#6	AGTGGGGCTGTCTGCGACAA	chr11	793219	27414	0	0,00	13965	2	0,01
OFT#7	TGTGAACCACACTGTGACAA	chrX	126483786	30402	0	0,00	7328	0	0,00
OFT#8	AGTGTGCTCCACTGTGACAA	chr18	23078807	632	0	0,00	724	0	0,00
OFT#9	CGTGGGCCAGCCTGGGACAA	chrX	153625648	19552	2	0,01	8569	0	0,00
OFT#10	AGAGAGCCACACTGAGACAG	chr5	133765919	64679	0	0,00	34737	13	0,04
OFT#11	AGTGGACTAAACTGGGACAA	chr20	43384613	7850	0	0,00	7125	0	0,00
OFT#12	AGTTGGGTACACTGCTACAA	chr16	126014	7529	0	0,00	6463	0	0,00
OFT#13	AGTGAGTCATACTGCTACAA	chr3	187901508	10751	2	0,02	8846	2	0,02
OFT#14	AGCTGACCACACTGCCACAA	chrX	150151366	5960	0	0,00	6062	2	0,03
OFT#15	AGTCTCCCACACTGCCACAA	chr2	12678638	10584	0	0,00	9012	0	0,00
OFT#16	TGTGAGCCACATTGCTACAA	chr19	15734255	6994	0	0,00	7762	0	0,00
OFT#17	AGGGGGGCACACAGCGACCA	chr1	205676263	5157	0	0,00	4637	0	0,00
OFT#18	AGAGGGGCACACTGAGACAC	chr16	4242661	702	0	0,00	977	0	0,00
OFT#19	AGTGGTACCCACTGGGACAA	chr9	82562622	1705	0	0,00	3451	0	0,00
OFT#20	TGTGGTCCACACAGCCACAA	chr15	53624548	11956	0	0,00	9876	2	0,02
OFT#21	AGTGAACCACACTGCTTCAA	chr7	63192705	6343	0	0,00	3906	0	0,00
OFT#22	AGTGAACCACACTGCTTCAA	chr4	110449855	658	0	0,00	668	0	0,00
OFT#23	AGTGAGCCATACTGTAACAA	chr7	79273708	2718	0	0,00	5357	0	0,00
OFT#24	AGTGGTCCACCCTGAGACAC	chr17	5443555	3568	0	0,00	3583	0	0,00
OFT#25	AGTGGGTCCCACAGGGACAA	chr10	69889535	693	0	0,00	1352	0	0,00
OFT#26	ATTGGTCCACACTGTGAGAA	chr4	140034020	9981	0	0,00	10159	0	0,00
OFT#27	TGTGGGCCACAGTGCCCCAA	chr11	97908565	16731	10	0,06	12504	6	0,05

				A673 LVCas9_NT			A673 LVCas9_EF		
OFF Target **A673**	Sequences (5'-3')	Chromosome	Position	Total reads	Modified Reads	Off-target (%)	Total reads	Modified Reads	Off-target (%)
sgE3	TGGTTGCACAGTAAGTGGCG								
OFT#1	TGATTGCACTGTAAGTGGCC	chr5	78568232	1542	0	0,00	16010	1	0,01
OFT#2	TAGGTGCTCAGTAAGTGGCT	chr10	35792862	21610	0	0,00	32765	0	0,00
OFT#3	CGACTTCACAGTAAGTGGCG	chr18	74446599	26865	0	0,00	6309	0	0,00
OFT#4	AGGTAGCGCAGTTAGTGGCG	chr9	14347518	12091	0	0,00	19888	0	0,00
OFT#5	AGGTAGAACAGTAAGTGGCA	chr1	194593114	4982	0	0,00	28486	4	0,01
OFT#6	TAGTTGTTCAGTAAGTGGCA	chr1	11805340	31235	2	0,01	60048	2	0,00
OFT#7	GGGTAGCAGAGGAAGTGGCG	chr19	32779534	42436	4	0,01	62331	2	0,00
OFT#8	TGGATGCTGAGTAAGTGGCC	chrX	115433334	36814	0	0,00	60048	2	0,00
OFT#9	TGCTTGCAAGGTAAGTGGCC	chr2	162011934	34218	9	0,03	30383	0	0,00
OFT#10	TGGTGGCACAGTAACTGGCC	chr4	122234874	12117	0	0,00	7373	0	0,00
OFT#11	AGGGTGCAAAGTGAGTGGCG	chr5	178332117	1251	0	0,00	481	0	0,00
OFT#12	TGGTGGCCCAGGAAGTGGCC	chr11	15652849	2906	0	0,00	1355	0	0,00
OFT#13	AAGTTGCACAGTCAGTGGCA	chr12	117053997	4062	0	0,00	2257	0	0,00
OFT#14	GGGGTGCACAGCAAGTGGTG	chr3	172710847	5654	0	0,00	2741	0	0,00
OFT#15	AGATTGCACGGTAAGGGGCG	chr21	45185099	2383	0	0,00	468	0	0,00
OFT#16	TGGCTGGACAGTCAGTGGCT	chr4	27092448	24764	0	0,00	20977	0	0,00
OFT#17	TGGCTGCAGAGGAAGTGGGG	chr12	120653158	10689	10	0,09	6123	8	0,13
OFT#18	TGTTTGCACAAGAAGTGGTG	chr3	1052161	14458	0	0,00	8504	0	0,00
OFT#19	TTATTGCACAGTAGGTGGCA	chr5	84015337	12655	0	0,00	8255	0	0,00
OFT#20	TGCTTGCATAGTAAATGGCA	chrX	20173722	9452	7	0,07	4448	2	0,04
OFT#21	AGGTTGCATAGTAAGTGTCA	chr11	74340172	10112	0	0,00	6328	0	0,00
sgF8	AGTGGGCCACACTGCGACAA								
OFT#1	GATTGGCCACACTGTGACAA	chr9	124911364	35337	4	0,01	46550	7	0,02
OFT#2	GGCGGGGCACACAGCGACAA	chr1	20716449	7774	0	0,00	1324	0	0,00
OFT#3	AGTGAGGAACACTGCGGCAA	chr6	30452057	40653	4	0,01	9737	2	0,02
OFT#4	TGTGGGCCAGGCTGCGGCAA	chr14	58819512	32163	0	0,00	40165	0	0,00
OFT#5	AGTGGGCTAGCCTGCGACAG	chr10	3167786	39513	0	0,00	8248	9	0,11
OFT#6	AGTGGGGCTGTCTGCGACAA	chr11	793219	21076	2	0,01	2091	0	0,00
OFT#7	TGTGAACCACACTGTGACAA	chrX	126483786	14647	0	0,00	22616	0	0,00
OFT#8	AGTGTGCTCCACTGTGACAA	chr18	23078807	2820	0	0,00	3019	0	0,00
OFT#9	CGTGGGCCAGCCTGGGACAA	chrX	153625648	10867	0	0,00	6433	0	0,00
OFT#10	AGAGAGCCACACTGAGACAG	chr5	133765919	26725	9	0,03	5907	0	0,00
OFT#11	AGTGGACTAAACTGGGACAA	chr20	43384613	10597	2	0,02	6448	0	0,00
OFT#12	AGTTGGGTACACTGCTACAA	chr16	126014	9917	0	0,00	5297	0	0,00
OFT#13	AGTGAGTCATACTGCTACAA	chr3	187901508	17549	3	0,02	12150	4	0,03
OFT#14	AGCTGACCACACTGCCACAA	chrX	150151366	17724	0	0,00	11574	0	0,00
OFT#15	AGTCTCCCACACTGCCACAA	chr2	12678638	17708	0	0,00	11698	0	0,00
OFT#16	TGTGAGCCACATTGCTACAA	chr19	15734255	11445	0	0,00	5884	0	0,00
OFT#17	AGGGGGGCACACAGCGACCA	chr1	205676263	7232	0	0,00	3751	0	0,00
OFT#18	AGAGGGGCACACTGAGACAC	chr16	4242661	1872	0	0,00	701	0	0,00
OFT#19	AGTGGTACCCACTGGGACAA	chr9	82562622	4342	3	0,07	2057	0	0,00
OFT#20	TGTGGTCCACACAGCCACAA	chr15	53624548	18418	0	0,00	13442	0	0,00
OFT#21	AGTGAACCACACTGCTTCAA	chr7	63192705	7482	0	0,00	5452	0	0,00
OFT#22	AGTGAACCACACTGCTTCAA	chr4	110449855	988	0	0,00	534	0	0,00
OFT#23	AGTGAGCCATACTGTAACAA	chr7	79273708	7383	0	0,00	3521	0	0,00
OFT#24	AGTGGTCCACCCTGAGACAC	chr17	5443555	5434	0	0,00	2397	0	0,00
OFT#25	AGTGGGTCCCACAGGGACAA	chr10	69889535	4203	0	0,00	1090	0	0,00
OFT#26	ATTGGTCCACACTGTGAGAA	chr4	140034020	12050	0	0,00	17153	0	0,00
OFT#27	TGTGGGCCACAGTGCCCCAA	chr11	97908565	30127	19	0,06	25186	15	0,06

OFF Target RD-ES	Sequences (5'-3')	Chromosome	Position	RD-ES LVCas9_NT			RD-ES LVCas9_EF		
				Total reads	Modified Reads	Off-target (%)	Total reads	Modified Reads	Off-target (%)
sgE3	TGGTTGCACAGTAAGTGGCG								
OFT#1	TGATTGCACTGTAAGTGGCC	chr5	78568232	14507	0	0,00	22462	3	0,01
OFT#2	TAGGTGCTCAGTAAGTGGCT	chr10	35792862	32140	0	0,00	22019	5	0,02
OFT#3	CGACTTCACAGTAAGTGGCG	chr18	74446599	35951	4	0,01	36436	0	0,00
OFT#4	AGGTAGCGCAGTTAGTGGCG	chr9	14347518	13125	0	0,00	16382	5	0,03
OFT#5	AGGTAGAACAGTAAGTGGCA	chr1	194593114	5559	0	0,00	27713	2	0,01
OFT#6	TAGTTGTTCAGTAAGTGGCA	chr1	11805340	57216	0	0,00	53673	3	0,01
OFT#7	GGGTAGCAGAGGAAGTGGCG	chr19	32779534	27412	0	0,00	40461	0	0,00
OFT#8	TGGATGCTGAGTAAGTGGCC	chrX	115433334	1017	0	0,00	59512	2	0,00
OFT#9	TGCTTGCAAGGTAAGTGGCC	chr2	162011934	32978	0	0,00	40946	0	0,00
OFT#10	TGGTGGCACAGTAACTGGCC	chr4	122234874	2767	0	0,00	5461	0	0,00
OFT#11	AGGGTGCAAAGTGAGTGGCG	chr5	178332117	127	0	0,00	487	0	0,00
OFT#12	TGGTGGCCCAGGAAGTGGCC	chr11	15652849	576	0	0,00	1381	0	0,00
OFT#13	AAGTTGCACAGTCAGTGGCA	chr12	117053997	789	0	0,00	1910	0	0,00
OFT#14	GGGGTGCACAGCAAGTGGTG	chr3	172710847	800	0	0,00	2133	0	0,00
OFT#15	AGATTGCACGGTAAGGGGCG	chr21	45185099	99	0	0,00	185	0	0,00
OFT#16	TGGCTGGACAGTCAGTGGCT	chr4	27092448	12561	0	0,00	20207	0	0,00
OFT#17	TGGCTGCAGAGGAAGTGGGG	chr12	120653158	1538	2	0,13	4500	4	0,09
OFT#18	TGTTTGCACAAGAAGTGGTG	chr3	1052161	10509	0	0,00	18370	0	0,00
OFT#19	TTATTGCACAGTAGGTGGCA	chr5	84015337	3280	0	0,00	5304	2	0,04
OFT#20	TGCTTGCATAGTAAATGGCA	chrX	20173722	3598	0	0,00	5809	0	0,00
OFT#21	AGGTTGCATAGTAAGTGTCA	chr11	74340172	3200	0	0,00	7076	0	0,00
sgF8	AGTGGGCCACACTGCGACAA								
OFT#1	GATTGGCCACACTGTGACAA	chr9	124911364	23984	6	0,03	24215	0	0,00
OFT#2	GGCGGGGCACACAGCGACAA	chr1	20716449	2140	0	0,00	42322	4	0,01
OFT#3	AGTGAGGAACACTGCGGCAA	chr6	30452057	86311	0	0,00	55558	5	0,01
OFT#4	TGTGGGCCAGGCTGCGGCAA	chr14	58819512	185	0	0,00	31686	10	0,03
OFT#5	AGTGGGCTAGCCTGCGACAG	chr10	3167786	22928	0	0,00	27899	0	0,00
OFT#6	AGTGGGGCTGTCTGCGACAA	chr11	793219	9604	0	0,00	30315	2	0,01
OFT#7	TGTGAACCACACTGTGACAA	chrX	126483786	22152	10	0,05	14962	0	0,00
OFT#8	AGTGTGCTCCACTGTGACAA	chr18	23078807	13586	1	0,01	56914	0	0,00
OFT#9	CGTGGGCCAGCCTGGGACAA	chrX	153625648	14991	0	0,00	20784	0	0,00
OFT#10	AGAGAGCCACACTGAGACAG	chr5	133765919	40508	8	0,02	30937	0	0,00
OFT#11	AGTGGACTAAACTGGGACAA	chr20	43384613	4241	0	0,00	8859	2	0,02
OFT#12	AGTTGGGTACACTGCTACAA	chr16	126014	2983	0	0,00	7809	2	0,03
OFT#13	AGTGAGTCATACTGCTACAA	chr3	187901508	8218	0	0,00	13338	0	0,00
OFT#14	AGCTGACCACACTGCCACAA	chrX	150151366	5587	0	0,00	9534	0	0,00
OFT#15	AGTCTCCCACACTGCCACAA	chr2	12678638	4918	0	0,00	7668	0	0,00
OFT#16	TGTGAGCCACATTGCTACAA	chr19	15734255	2594	0	0,00	5988	0	0,00
OFT#17	AGGGGGGCACACAGCGACCA	chr1	205676263	1555	0	0,00	4040	0	0,00
OFT#18	AGAGGGGCACACTGAGACAC	chr16	4242661	220	0	0,00	675	0	0,00
OFT#19	AGTGGTACCCACTGGGACAA	chr9	82562622	714	0	0,00	984	0	0,00
OFT#20	TGTGGTCCACACAGCCACAA	chr15	53624548	5510	0	0,00	9479	0	0,00
OFT#21	AGTGAACCACACTGCTTCAA	chr7	63192705	9591	0	0,00	15813	0	0,00
OFT#22	AGTGAACCACACTGCTTCAA	chr4	110449855	140	0	0,00	266	0	0,00
OFT#23	AGTGAGCCATACTGTAACAA	chr7	79273708	1154	0	0,00	1911	0	0,00
OFT#24	AGTGGTCCACCCTGAGACAC	chr17	5443555	1544	0	0,00	3492	0	0,00
OFT#25	AGTGGGTCCCACAGGGACAA	chr10	69889535	291	0	0,00	650	0	0,00
OFT#26	ATTGGTCCACACTGTGAGAA	chr4	140034020	4620	0	0,00	7872	0	0,00
OFT#27	TGTGGGCCACAGTGCCCCAA	chr11	97908565	19393	6	0,03	29281	4	0,01

OFF Target Xenograft	Sequences (5'-3')	Chromosome	Position	Xenograft AdCas9_NT			Xenograft AdCas9_EF		
				Total reads	Modified Reads	Off-target (%)	Total reads	Modified Reads	Off-target (%)
sgE3	TGGTTGCACAGTAAGTGGCG								
OFT#2	TAGGTGCTCAGTAAGTGGCT	chr10	35792862	9301	0	0,00	1032	0	0,00
OFT#6	TAGTTGTTCAGTAAGTGGCA	chr1	11805340	2807	0	0,00	273	0	0,00
OFT#7	GGGTAGCAGAGGAAGTGGCG	chr19	32779534	10521	0	0,00	1875	0	0,00
OFT#8	TGGATGCTGAGTAAGTGGCC	chrX	115433334	10954	6	0,05	1614	0	0,00
OFT#10	TGGTGGCACAGTAACTGGCC	chr4	122234874	11721	2	0,02	1580	0	0,00
OFT#11	AGGGTGCAAAGTGAGTGGCG	chr5	178332117	826	0	0,00	20	0	0,00
OFT#12	TGGTGGCCCAGGAAGTGGCC	chr11	15652849	7420	0	0,00	1068	0	0,00
OFT#13	AAGTTGCACAGTCAGTGGCA	chr12	117053997	6617	0	0,00	1448	0	0,00
OFT#14	GGGGTGCACAGCAAGTGGTG	chr3	172710847	3645	0	0,00	258	0	0,00
OFT#16	TGGCTGGACAGTCAGTGGCT	chr4	27092448	15015	0	0,00	2497	0	0,00
OFT#17	TGGCTGCAGAGGAAGTGGGG	chr12	120653158	12434	12	0,10	1931	0	0,00
OFT#18	TGTTTGCACAAGAAGTGGTG	chr3	1052161	9821	0	0,00	1419	0	0,00
OFT#19	TTATTGCACAGTAGGTGGCA	chr5	84015337	7918	0	0,00	907	0	0,00
OFT#20	TGCTTGCATAGTAAATGGCA	chrX	20173722	6735	0	0,00	835	0	0,00
OFT#21	AGGTTGCATAGTAAGTGTCA	chr11	74340172	7871	0	0,00	1167	0	0,00
sgF8	AGTGGGCCACACTGCGACAA								
OFT#1	GATTGGCCACACTGTGACAA	chr9	124911364	15685	4	0,03	3236	0	0,00
OFT#2	GGCGGGGCACACAGCGACAA	chr1	20716449	283	0	0,00	2	0	0,00
OFT#7	TGTGAACCACACTGTGACAA	chrX	126483786	6937	0	0,00	774	0	0,00
OFT#11	AGTGGACTAAACTGGGACAA	chr20	43384613	11620	3	0,03	2014	0	0,00
OFT#12	AGTTGGGTACACTGCTACAA	chr16	126014	10267	2	0,02	1307	0	0,00
OFT#13	AGTGAGTCATACTGCTACAA	chr3	187901508	11430	2	0,02	1716	0	0,00
OFT#14	AGCTGACCACACTGCCACAA	chrX	150151366	9494	4	0,04	1560	0	0,00
OFT#15	AGTCTCCCACACTGCCACAA	chr2	12678638	10764	0	0,00	1559	0	0,00
OFT#16	TGTGAGCCACATTGCTACAA	chr19	15734255	9449	0	0,00	1319	0	0,00
OFT#17	AGGGGGGCACACAGCGACCA	chr1	205676263	6058	0	0,00	720	0	0,00
OFT#18	AGAGGGGCACACTGAGACAC	chr16	4242661	6031	2	0,03	833	0	0,00
OFT#19	AGTGGTACCCACTGGGACAA	chr9	82562622	2643	2	0,08	173	0	0,00
OFT#20	TGTGGTCCACACAGCCACAA	chr15	53624548	10810	0	0,00	1509	0	0,00
OFT#21	AGTGAACCACACTGCTTCAA	chr7	63192705	4106	0	0,00	537	0	0,00
OFT#22	AGTGAACCACACTGCTTCAA	chr4	110449855	496	0	0,00	32	0	0,00
OFT#23	AGTGAGCCATACTGTAACAA	chr7	79273708	4455	0	0,00	387	0	0,00
OFT#24	AGTGGTCCACCCTGAGACAC	chr17	5443555	8528	0	0,00	1375	0	0,00
OFT#25	AGTGGGTCCCACAGGGACAA	chr10	69889535	400	0	0,00	11	0	0,00
OFT#26	ATTGGTCCACACTGTGAGAA	chr4	140034020	11685	0	0,00	1606	0	0,00
OFT#27	TGTGGGCCACAGTGCCCCAA	chr11	97908565	14218	8	0,06	1565	0	0,00

				PDX LVCas9_NT			PDX LVCas9_EF		
OFF Target **PDX**	Sequences (5'-3')	Chromosome	Position	Total reads	Modified Reads	Off-target (%)	Total reads	Modified Reads	Off-target (%)
sgE3	TGGTTGCACAGTAAGTGGCG								
OFT#2	TAGGTGCTCAGTAAGTGGCT	chr10	35792862	7602	0	0,00	6929	0	0,00
OFT#6	TAGTTGTTCAGTAAGTGGCA	chr1	11805340	924	0	0,00	1603	0	0,00
OFT#7	GGGTAGCAGAGGAAGTGGCG	chr19	32779534	9678	0	0,00	8040	0	0,00
OFT#8	TGGATGCTGAGTAAGTGGCC	chrX	115433334	6157	0	0,00	4321	2	0,05
OFT#10	TGGTGGCACAGTAACTGGCC	chr4	122234874	8351	0	0,00	7924	0	0,00
OFT#11	AGGGTGCAAAGTGAGTGGCG	chr5	178332117	324	0	0,00	364	0	0,00
OFT#12	TGGTGGCCCAGGAAGTGGCC	chr11	15652849	7788	0	0,00	6242	0	0,00
OFT#13	AAGTTGCACAGTCAGTGGCA	chr12	117053997	2906	0	0,00	4158	0	0,00
OFT#14	GGGGTGCACAGCAAGTGGTG	chr3	172710847	2607	0	0,00	1709	0	0,00
OFT#16	TGGCTGGACAGTCAGTGGCT	chr4	27092448	14261	0	0,00	11120	0	0,00
OFT#17	TGGCTGCAGAGGAAGTGGGG	chr12	120653158	10512	17	0,16	9081	16	0,18
OFT#18	TGTTTGCACAAGAAGTGGTG	chr3	1052161	9582	0	0,00	8164	0	0,00
OFT#19	TTATTGCACAGTAGGTGGCA	chr5	84015337	7177	0	0,00	6413	0	0,00
OFT#20	TGCTTGCATAGTAAATGGCA	chrX	20173722	2242	0	0,00	2304	0	0,00
OFT#21	AGGTTGCATAGTAAGTGTCA	chr11	74340172	5603	2	0,04	4790	0	0,00
sgF8	AGTGGGCCACACTGCGACAA								
OFT#1	GATTGGCCACACTGTGACAA	chr9	124911364	14195	2	0,01	11348	2	0,02
OFT#2	GGCGGGGCACACAGCGACAA	chr1	20716449	38	0	0,00	41	0	0,00
OFT#7	TGTGAACCACACTGTGACAA	chrX	126483786	2869	0	0,00	2851	0	0,00
OFT#11	AGTGGACTAAACTGGGACAA	chr20	43384613	10245	0	0,00	8378	7	0,08
OFT#12	AGTTGGGTACACTGCTACAA	chr16	126014	7821	0	0,00	6167	0	0,00
OFT#13	AGTGAGTCATACTGCTACAA	chr3	187901508	9651	2	0,02	8681	0	0,00
OFT#14	AGCTGACCACACTGCCACAA	chrX	150151366	5512	0	0,00	4411	0	0,00
OFT#15	AGTCTCCCACACTGCCACAA	chr2	12678638	9409	0	0,00	7960	0	0,00
OFT#16	TGTGAGCCACATTGCTACAA	chr19	15734255	8968	0	0,00	7836	0	0,00
OFT#17	AGGGGGGCACACAGCGACCA	chr1	205676263	5129	0	0,00	3719	0	0,00
OFT#18	AGAGGGGCACACTGAGACAC	chr16	4242661	3376	0	0,00	4134	0	0,00
OFT#19	AGTGGTACCCACTGGGACAA	chr9	82562622	2012	0	0,00	1897	0	0,00
OFT#20	TGTGGTCCACACAGCCACAA	chr15	53624548	10195	0	0,00	8848	0	0,00
OFT#21	AGTGAACCACACTGCTTCAA	chr7	63192705	6175	0	0,00	6605	0	0,00
OFT#22	AGTGAACCACACTGCTTCAA	chr4	110449855	761	0	0,00	814	0	0,00
OFT#23	AGTGAGCCATACTGTAACAA	chr7	79273708	3332	30	0,90	3134	25	0,80
OFT#24	AGTGGTCCACCCTGAGACAC	chr17	5443555	3033	0	0,00	2601	0	0,00
OFT#25	AGTGGGTCCCACAGGGACAA	chr10	69889535	40	0	0,00	19	0	0,00
OFT#26	ATTGGTCCACACTGTGAGAA	chr4	140034020	10310	0	0,00	9064	0	0,00
OFT#27	TGTGGGCCACAGTGCCCCAA	chr11	97908565	11205	7	0,06	8219	4	0,05

				K562 LVCas9_NT			K562 LVCas9_BA		
OFF Target **K562**	sgRNA sequence (5'-3')	Chromosome	Position	Total reads	Modified Reads	Off-target (%)	Total reads	Modified Reads	Off-target (%)
sgB8.2	TATCCGAGGCACGTTAAGGG								
OFT#1	GTTCAGAGCCACGTTAAGGG	chr6	148680632	2529	0	0.0	4481	0	0.0
OFT#2	AACCAAAGGCACGTTAAGGG	chr8	74818262	407	0	0.0	230	0	0.0
OFT#3	TAGATGAGGCATGTTAAGGG	chr18	439005	5820	0	0.0	8109	2	0.0
OFT#4	TTTCTCAGGCAGGTTAAGGG	chr18	63520338	25320	0	0.0	32468	8	0.0
OFT#5	TAGCCGAATCATGTTAAGGG	chr2	61770649	1886	0	0.0	1102	0	0.0
sgA1.1	CACGAGGTTGACGCACCAGA								
OFT#1	CAGGAGGTGGAAGCACCAGA	chr3	53121575	346	0	0.0	2813	2	0.1
OFT#2	CATTGGGTTGACGCACCAGC	chr10	69632740	235	0	0.0	23683	7	0.0
OFT#3	GACCAGGTTTACACACCAGA	chr4	3645283	62046	10	0.0	637	0	0.0
OFT#4	GACCAGGTTGAGGCACCAGG	chr7	44101489	14262	2	0.0	353	0	0.0
OFT#5	CTCCAGGTTGATGCACCAGG	chr21	42109613	44893	3	0.0	2035	0	0.0

Supplementary Table 3. Indel analysis of the most probable off-target sites with the highest homology to the on-target sites by amplicon based next-generation sequencing analysis at day 7 post-transduction of hMSC, A673, RD-ES, xenograft (A673), PDX and K562 cells. On-target sequences are listed at the top of each panel. Differences in nucleotides with on-target sequence are shown in red.
