# Supplementary material for: In vivo CRISPR/Cas9 targeting of fusion oncogenes for selective elimination of cancer cells
Source: Nat Commun. 2020 Oct 8;11:5060. doi: 10.1038/s41467-020-18875-x (PMC7544871; doi:10.1038/s41467-020-18875-x)
Supplement: Supplementary file 3 — Reporting Summary [file 41467_2020_18875_MOESM3_ESM.pdf]

nature research

Corresponding author(s): Sandra Rodriguez-Perales
Raul Torres-Ruiz

Last updated by author(s): Aug 31, 2020

# Reporting Summary

Nature Research wishes to improve the reproducibility of the work that we publish. This form provides structure for consistency and transparency in reporting. For further information on Nature Research policies, see Authors & Referees and the Editorial Policy Checklist.

## Statistics

For all statistical analyses, confirm that the following items are present in the figure legend, table legend, main text, or Methods section.

| n/a | Confirmed | |
|---|---|---|
| ☐ | ☒ | The exact sample size (*n*) for each experimental group/condition, given as a discrete number and unit of measurement |
| ☐ | ☒ | A statement on whether measurements were taken from distinct samples or whether the same sample was measured repeatedly |
| ☐ | ☒ | The statistical test(s) used AND whether they are one- or two-sided<br>*Only common tests should be described solely by name; describe more complex techniques in the Methods section.* |
| ☒ | ☐ | A description of all covariates tested |
| ☒ | ☐ | A description of any assumptions or corrections, such as tests of normality and adjustment for multiple comparisons |
| ☐ | ☒ | A full description of the statistical parameters including central tendency (e.g. means) or other basic estimates (e.g. regression coefficient) AND variation (e.g. standard deviation) or associated estimates of uncertainty (e.g. confidence intervals) |
| ☐ | ☒ | For null hypothesis testing, the test statistic (e.g. *F*, *t*, *r*) with confidence intervals, effect sizes, degrees of freedom and *P* value noted<br>*Give P values as exact values whenever suitable.* |
| ☒ | ☐ | For Bayesian analysis, information on the choice of priors and Markov chain Monte Carlo settings |
| ☒ | ☐ | For hierarchical and complex designs, identification of the appropriate level for tests and full reporting of outcomes |
| ☒ | ☐ | Estimates of effect sizes (e.g. Cohen's *d*, Pearson's *r*), indicating how they were calculated |

*Our web collection on statistics for biologists contains articles on many of the points above.*

## Software and code

Policy information about availability of computer code

| | |
|---|---|
| Data collection | FACS DATA - BD FACSCanto™ platform<br>qPCR DATA - ABI-Prism7900HT Detection System<br>Sequencing data - Illumina MiSeq<br>Olympus AX70 microscope<br>Karyotype-Zeiss Axioplan microscope |
| Data analysis | Graph plotting and statistical analysis - Graphpad Prism v7<br>FACS analysis - Flowjo v10<br>sgRNA design-Benchling [Biology Software]. (2018). Retrieved from https://benchling.com<br>Cas-OFFinder (http://www.rgenome.net/cas-offinder) v2.4<br>Snap gene v2.3.2<br>Immunoassays-Cytovision v7.4 software (Leica Biosystem).<br>ImageJ v1.51p<br>Karyotype-Ikaros v5.20 karyotyping platform<br>aCGH-Cytogenomics v5.0<br>genome.ucsc.edu (2018); UCSC Genome Browser assembly ID: hg38 |

For manuscripts utilizing custom algorithms or software that are central to the research but not yet described in published literature, software must be made available to editors/reviewers. We strongly encourage code deposition in a community repository (e.g. GitHub). See the Nature Research guidelines for submitting code & software for further information.

## Data

Policy information about availability of data

All manuscripts must include a data availability statement. This statement should provide the following information, where applicable:

- Accession codes, unique identifiers, or web links for publicly available datasets
- A list of figures that have associated raw data
- A description of any restrictions on data availability

Data that support the findings of the present manuscript are available from the corresponding authors upon reasonable request.

# Field-specific reporting

Please select the one below that is the best fit for your research. If you are not sure, read the appropriate sections before making your selection.

☒ Life sciences ☐ Behavioural & social sciences ☐ Ecological, evolutionary & environmental sciences

For a reference copy of the document with all sections, see nature.com/documents/nr-reporting-summary-flat.pdf

# Life sciences study design

All studies must disclose on these points even when the disclosure is negative.

| | |
|---|---|
| Sample size | For each experiment, a sample size was chosen to obtain sufficient number of experiments and samples to calculate statistical significance. Sample sizes were chosen based on previous publications. No statistical test was performed to predetermine sample size. |
| Data exclusions | No collected data were excluded from the analysis. |
| Replication | Data presented were replicated in at least 3 independent experiments with similar results |
| Randomization | Allocation to samples or mice to the experimental groups was random. |
| Blinding | For practical reasons investigators were not blinded to treatment administration in mice experiments. However, for all other analysis samples were identify with an ID number that do not contains information about treatment administered (in a blinded manner). Treatment assigned to mice was random. Data collection for all automated experiments (e.g. flow cytometry, sequencing etc.) and data interpretation was based on appropriate controls. |

# Reporting for specific materials, systems and methods

We require information from authors about some types of materials, experimental systems and methods used in many studies. Here, indicate whether each material, system or method listed is relevant to your study. If you are not sure if a list item applies to your research, read the appropriate section before selecting a response.

### Materials & experimental systems

| n/a | Involved in the study | |
|---|---|---|
| ☐ | ☒ | Antibodies |
| ☐ | ☒ | Eukaryotic cell lines |
| ☒ | ☐ | Palaeontology |
| ☐ | ☒ | Animals and other organisms |
| ☒ | ☐ | Human research participants |
| ☒ | ☐ | Clinical data |

### Methods

| n/a | Involved in the study | |
|---|---|---|
| ☒ | ☐ | ChIP-seq |
| ☐ | ☒ | Flow cytometry |
| ☒ | ☐ | MRI-based neuroimaging |

## Antibodies

Antibodies used

anti-FLI1 mouse monoclonal (G146-254) antibody (1/400; BD Pharmigen; ref:554267)
anti-GAPDH mouse monoclonal antibody (6C5) (1/200; Abcam, ref:ab8245)
HRP-conjugated with goat anti-mouse IgG (1/1000; Abcam, ref:ab205719)
anti-caspase-3 rabbit (C92-605) antibody (1/200; BD. ref:559565)
anti-Ki-67 Mouse Monoclonal (MIB-1) antibody (1:1.000; DAKO; ref:IR626)
anti-CD45 antibody Rabbit Monoclonal (D3F8Q) antibody(1:200; Cell Signalling Technology, ref:70257)
anti-GFP antibody Rabbit Monoclonal (D5.1) antibody(1:200; Cell Signalling Technology, ref:2956)
anti-C3-cleaved caspase-3 Rabbit Polyclonal antibody ( 1:800; Cell Signalling Technology; ref:9661)
anti-Cas9 (7A9-3A3) rabbit polyclonal antibody (1:1000; Cell Signalling Technology; ref:14697)

| | |
|---|---|
| | Alexa Fluor-594-conjugated secondary antibody (1/500; ThermoFisher Sci, ref:A32740) |
| Validation | All the available information on antibody validation can be found by searching the manufacturer's websites.<br>anti-FLI1 mouse monoclonal (G146-254) antibody validated in western blot analysis of Lysate from Jurkat cells at differnt concentrations. Fli-1 is identified as a band of ~50 kDa.<br>anti-GAPDH mouse monoclonal antibody validated in western blot analysis of extract from various cell lines. Human Burkitt's lymphoma cell line, HeLa cell line, A431 cell line, HEK-293 obtaining a band at 37kDa.<br>anti-caspase-3 rabbit (C92-605) antibody. This antibody has been reported to specifically recognize the active form of caspase-3 in human and mouse cells. It has been used in different research articles for immunofluorescence such as Lloyd, A. F., Davies, C. L., et al., Nature Neuroscience 2019 or Floyd, S. R., Pacold, M. E., et al., Nature 2013<br>anti-Ki-67 Mouse Monoclonal (MIB-1) antibody validated in immunohistochemistry analysis of different paraffin-embedded specimens such as Burkitt lymphoma/leukemia, colon adenocarcinoma, soft-tissue sarcoma, prostatic adenocarcinoma and breast carcinoma.<br>anti-CD45 antibody Rabbit Monoclonal (D3F8Q) antibody validated in immunohistochemistry analysis of paraffin-embedded 4T1 mammary tumor.<br>anti-GFP antibody Rabbit Monoclonal (D5.1) antibody validated in immunohistochemistry analysis of paraffin-embedded HCC827 untransfected or GFP-transfected cells.<br>anti-C3-cleaved caspase-3 Rabbit Polyclonal for validated in immunohistochemistry analysis of paraffin-embedded human tonsil and in paraffin-embedded Jurkat cells, untreated or etoposide-treated).<br>anti-Cas9 (7A9-3A3) rabbit polyclonal antibody validated in immunohistochemistry analysis of Nprl2-deficient 293 (positive) and untreated 293 (negative) cell pellets using Cas9 (7A9-3A3) Mouse mAb Nprl2 expression was knocked out in the Nprl2-deficient cells by transient transfection of Cas9 and Nprl2-specific guide sequences. |

## Eukaryotic cell lines

Policy information about cell lines

| | |
|---|---|
| Cell line source(s) | HEK293T/17, A673, hMSC, U2OS, and K562 cells were purchased from the American Type Culture Collection (ATCC)<br>RD-ES cells (ATCC HTB-166) were a gift from Dr. Javier Alonso (Instituto Salud Carlos III, Madrid). hCD34+ were obtained from umbilical cord blood of healthy donors after informed consent was obtained and after approval by the Cord Blood Bank Transfusion Center of the Community of Madrid. |
| Authentication | Cell lines from ATCC have been thoroughly tested and authenticated by STR profile |
| Mycoplasma contamination | All cell lines were negative for mycoplasma test |
| Commonly misidentified lines (See ICLAC register) | no commonly misidentified cell lines were used in the study |

## Animals and other organisms

Policy information about studies involving animals; ARRIVE guidelines recommended for reporting animal research

| | |
|---|---|
| Laboratory animals | Mice used were 8-week-old athymic nude (Hds:Athymic Nude- Foxn1 nu) female mice (Charles River). Mice were housed in the specific pathogen-free animal house of the Spanish National Cancer Research Centre under conditions in accordance with the recommendations of the Federation of European Laboratory Animal Science Associations (FELASA). |
| Wild animals | No wild animals were used in these studies |
| Field-collected samples | No field collected samples were used in these studies |
| Ethics oversight | All experiments with mice met the Animal Welfare guidelines and were performed in accordance to protocols approved by the Ethics Committee of the Instituto de Salud Carlos III. |

Note that full information on the approval of the study protocol must also be provided in the manuscript.

## Flow Cytometry

### Plots

Confirm that:

☒ The axis labels state the marker and fluorochrome used (e.g. CD4-FITC).

☒ The axis scales are clearly visible. Include numbers along axes only for bottom left plot of group (a 'group' is an analysis of identical markers).

☒ All plots are contour plots with outliers or pseudocolor plots.

☒ A numerical value for number of cells or percentage (with statistics) is provided.

### Methodology

| | |
|---|---|
| Sample preparation | For Apoptotic SubG1 cell detection - Exponentially growing transduced cells were pelleted by centrifugation, washed with PBS |

| | |
|---|---|
| Sample preparation | (Sigma), resuspended in ice-cold 70% ethanol (Sigma) in PBS for fixation, and were maintained at 4°C for 24 h. Prior to analysis, fixed cells were washed with phosphate-citrate buffer (Sigma–Aldrich) and incubated with 200ul of 50ug/ml propidium iodide staining solution (Sigma-Aldrich) containing 100 µg/ml of RNase (Qiagen) for 30 min. Cellular DNA content was analysed on a BD FACSCanto™ platform using FlowJo® software. |
| Instrument | FACS Canto II, Beckton-Dickinson) |
| Software | Flowjo software v10 |
| Cell population abundance | Flow cytometry was used for quantification analysis only, no post sorting fractions were collected |
| Gating strategy | Forward scatter vs PI signal; PI histogram. |

☒ Tick this box to confirm that a figure exemplifying the gating strategy is provided in the Supplementary Information.
